# Genomic and transcriptomic features of androgen receptor signaling inhibitor resistance in metastatic castration-resistant prostate cancer

**DOI:** 10.1172/JCI178604

**Published:** 2024-08-13

**Authors:** Xiaolin Zhu, Tatyanah Farsh, Daniël Vis, Ivan Yu, Haolong Li, Tianyi Liu, Martin Sjöström, Raunak Shrestha, Jeroen Kneppers, Tesa Severson, Meng Zhang, Arian Lundberg, Thaidy Moreno Rodriguez, Alana S. Weinstein, Adam Foye, Niven Mehra, Rahul R. Aggarwal, Andries M. Bergman, Eric J. Small, Nathan A. Lack, Wilbert Zwart, David A. Quigley, Michiel S. van der Heijden, Felix Y. Feng

**Affiliations:** 1 Helen Diller Family Comprehensive Cancer Center, UCSF, San Francisco, California, USA.; 2 Division of Hematology and Oncology, Department of Medicine, UCSF, San Francisco, California, USA.; 3 Department of Radiation Oncology, UCSF, San Francisco, California, USA.; 4 Division of Molecular Carcinogenesis, Netherlands Cancer Institute, Amsterdam, Netherlands.; 5 Vancouver Prostate Centre, University of British Columbia, Vancouver, British Columbia, Canada.; 6 Division of Oncogenomics, Oncode Institute, Netherlands Cancer Institute, Amsterdam, Netherlands.; 7 Department of Urology, UCSF, San Francisco, California, USA.; 8 Department of Medical Oncology, Radboud University Medical Center, Nijmegen, Netherlands.; 9 Koç University School of Medicine, Istanbul, Turkey.; 10 Koç University Research Center for Translational Medicine (KUTTAM), Istanbul, Turkey.

**Keywords:** Oncology, Prostate cancer

## Abstract

**BACKGROUND:**

Androgen receptor signaling inhibitors (ARSIs) have improved outcomes for patients with metastatic castration-resistant prostate cancer (mCRPC), but their clinical benefit is limited by treatment resistance.

**METHODS:**

To investigate the mechanisms of ARSI resistance, we analyzed the whole-genome (*n* = 45) and transcriptome (*n* = 31) sequencing data generated from paired metastatic biopsies obtained before initiation of first-line ARSI therapy for mCRPC and after radiographic disease progression. We investigated the effects of genetic and pharmacologic modulation of SSTR1 in 22Rv1 cells, a representative mCRPC cell line.

**RESULTS:**

We confirmed the predominant role of tumor genetic alterations converging on augmenting androgen receptor (AR) signaling and the increased transcriptional heterogeneity and lineage plasticity during the emergence of ARSI resistance. We further identified amplifications involving a putative enhancer downstream of the AR and transcriptional downregulation of *SSTR1*, encoding somatostatin receptor 1, in ARSI-resistant tumors. We found that patients with *SSTR1*-low mCRPC tumors derived less benefit from subsequent ARSI therapy in a retrospective cohort. We showed that SSTR1 was antiproliferative in 22Rv1 cells and that the FDA-approved drug pasireotide suppressed 22Rv1 cell proliferation.

**CONCLUSION:**

Our findings expand the knowledge of ARSI resistance and point out actionable next steps, exemplified by potentially targeting SSTR1, to improve patient outcomes.

**FUNDING:**

National Cancer Institute (NCI), NIH; Prostate Cancer Foundation; Conquer Cancer, American Society of Clinical Oncology Foundation; UCSF Benioff Initiative for Prostate Cancer Research; Netherlands Cancer Institute.

## Abstract 2

Genomic analysis of paired metastatic prostate cancer biopsies unveils resistance mechanisms to novel hormonal therapies, presenting opportunities for innovative clinical interventions.

## Introduction

Metastatic castration-resistant prostate cancer (mCRPC) is the lethal form of prostate cancer (PCa) (1). Androgen receptor signaling inhibitors (ARSIs), such as abiraterone and enzalutamide, have improved outcomes for patients with mCRPC and hormone-sensitive prostate cancer (HSPC) (1). However, PCa invariably develops resistance to ARSIs, leaving patients with limited treatment options following disease progression (2). With the increasing use of ARSIs in PCa (3), understanding and overcoming ARSI resistance are key to improving patient outcomes.

Studies of preclinical models and patient samples have revealed recurring mechanisms of resistance to androgen deprivation therapy (ADT) and ARSIs. These mechanisms can be broadly grouped by their relationships to the androgen receptor (AR), the primary driver and drug target of PCa (2). AR-dependent mechanisms typically originate in the PCa genome and manifest as amplifications of *AR* (4) and its upstream enhancer (5–7) and gain-of-function mutations in the ligand binding domain (8). Conversely, non-AR-mediated mechanisms appear to be more heterogeneous (2) and may manifest as treatment-emergent evolution into AR-indifferent phenotypes such as small-cell neuroendocrine (NE) (9–14) or AR-negative, NE-negative (double-negative) PCa (11, 15–17). Nonetheless, how PCa becomes resistant to ARSIs remains inadequately understood, partially due to the difficulty of obtaining serial tissue biopsies through the treatment course, which would enable a direct search for treatment-emergent changes by contrasting progressive with baseline tumors in individual patients (18). To circumvent the need for tissue biopsies, recent studies serially sampled circulating tumor DNA (ctDNA) from patients and identified *AR* alterations as the main genetic driver of ARSI resistance (19, 20). Although liquid biopsy is an invaluable, noninvasive diagnostic tool, it complements rather than supplants tissue biopsy for a comprehensive understanding of resistance mechanisms.

To address this knowledge gap, we prospectively obtained metastatic biopsies from patients with mCRPC, before initiation of first-line abiraterone or enzalutamide and after radiographic progression on treatment. We generated and analyzed whole-genome sequencing (WGS) and RNA-Seq data on these paired mCRPC biopsies. A subset of these samples was analyzed and reported in our baseline WGS profiling of mCRPC (5) and earlier RNA-Seq studies of treatment-resistant PCa (13, 14). Here, we report the results of what we believe to be the largest systematic comparison of paired ARSI-resistant versus ARSI-naive mCRPC tumors to define the genomic and transcriptomic features of ARSI resistance.

## Results

**Figure 1:**
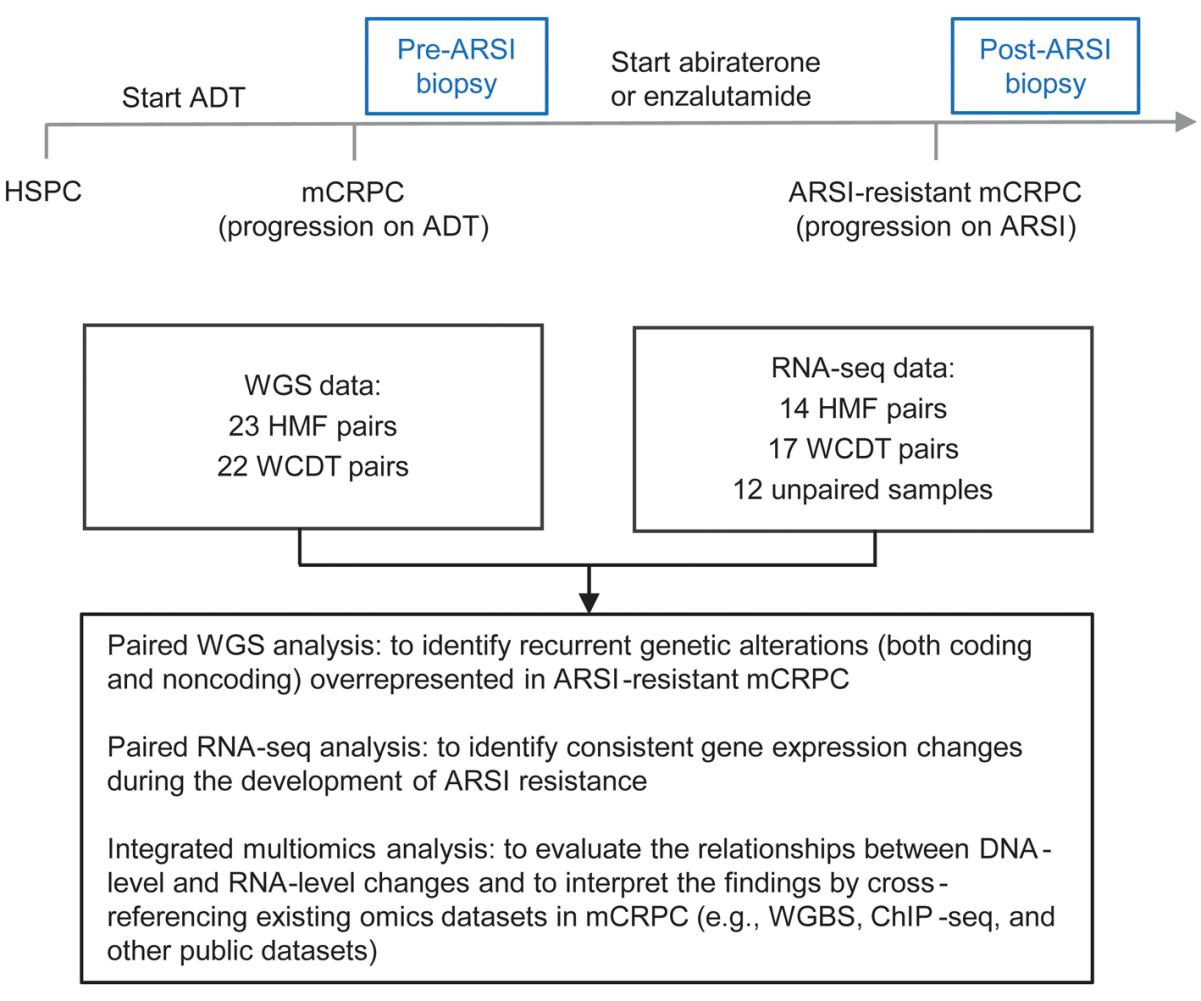
Overview of study design and analysis. Paired metastatic biopsies were obtained for patients with mCRPC before the initiation of an ARSI (pre-ARSI) and after radiographic progression on the ARSI (post-ARSI). Two cohorts (WCDT and HMF) were merged, with batch effects corrected as appropriate before downstream analysis. A total of 45 WGS pairs were analyzed, and for a subset of them, RNA-Seq data were successfully generated and analyzed.

We analyzed a total of 45 patients with paired tumor WGS data, 31 of whom had paired RNA-Seq data generated from the same tumor biopsies (Figure 1). Tumor WGS was performed to a mean coverage of 108X (range: 56–161X). The genomic landscape of the 45 paired biopsies is shown in Supplemental Figure 1; supplemental material available online with this article; https://doi.org/10.1172/JCI178604DS1 No significant difference in tumor purity, ploidy, tumor mutation burden, or structural variant (SV) burden was observed before or after ARSI therapy (Supplemental Figure 2). As expected, the change in variant allele frequency (VAF) of those single nucleotide variants (SNVs) and indels shared before and after ARSI therapy was strongly correlated with the change in tumor purity (Supplemental Figure 3). This observation led us to focus subsequent analyses on examining the mutations newly arising after ARSI (rather than comparing the pre- and post-ARSI VAFs of shared mutations) to avoid the confounding effect of tumor purity on VAF estimation.

### The AR locus is the primary substrate of convergent evolution under ARSI-induced selection.

To systematically search for genetic alterations associated with ARSI resistance, we first analyzed protein-coding mutations and ranked all genes by how frequently they harbored at least 1 qualifying mutation. A qualifying mutation was defined as an SNV or indel that met both criteria: (a) newly arising after ARSI and (b) predicted to change the protein sequence (including loss-of-function and missense variants). This analysis identified *AR* as the most frequently mutated gene, with at least 1 qualifying mutation observed in 4 of the 45 pairs (Supplemental Table 1). All qualifying mutations of *AR* are known hotspot mutations (L702H, H875Y, and T878A) in PCa (8). Among the 45 pairs, L702H and H875Y were found only after ARSI therapy (21), whereas T878A was observed both before and after ARSI (Supplemental Table 1). Prior studies reported that L702H conferred a growth advantage to PCa in the presence of glucocorticoids (22), and H875Y was detected in ctDNA after progression of the disease while the patient was on abiraterone or enzalutamide (23). Conversely, the effect of T878A on enzalutamide resistance remains undefined (8), and a recent study reported T878A in a patient after disease progression on abiraterone, who responded dramatically to darolutamide (24). We identified *AR* T878A in 3 pairs. In 2 of these pairs, it newly emerged (Hartwig Medical Foundation 017 [HMF-017]) or was preexisting but gained VAF after ARSI (19.6% → 48.8%, DTB-019); both patients were treated with abiraterone after the pre-ARSI biopsy followed by rapid progression (after 3 and 5.5 months, respectively) that prompted the post-ARSI biopsy, suggesting a role of T878A in abiraterone resistance. In the third pair (HMF-005), T878A was detected before ARSI but was paradoxically absent after ARSI (despite good sequencing coverage); the patient was treated for 17.6 months with enzalutamide after the pre-ARSI biopsy, before progression of the disease. Overall, our paired analysis suggests that *AR* hotspot mutations can arise at different time points through the course of hormonal therapy (8, 25). Aside from *AR*, we were unable to definitively identify another gene that recurrently gained new protein-coding mutations after ARSI treatment.

In addition to the qualifying mutations defined above, we listed all recurrent protein-coding mutations detected in pre-ARSI and post-ARSI tumors in Supplemental Tables 2 and 3, respectively. We found 4 genes with recurrent mutations before ARSI (*AKT1*, *AR*, *CDK12*, and *SPATA31E1*); the former 3 are known to mutate in PCa, and their mutations in our dataset are known to be pathogenic (*AKT1* E17K and *AR* T878A) or consistent with the known mechanisms of pathogenicity (the *CDK12* stop gain mutation causing loss of function). After ARSI, we found a total of 242 unique recurrent mutations (Supplemental Table 3), with *KMT2C* C988F being the most frequently mutated (detected in 3 post-ARSI samples, 2 from the same patient, DTB-176). This mutation is absent in primary PCa in The Cancer Genome Atlas (TCGA) (26) and is predicted to be benign as a germline mutation in ClinVar (27). Although *KMT2C* is frequently mutated in cancer, we believe further statistical and functional evidence is needed to clarify whether C988F is associated with ARSI resistance. All the other 241 mutations were mutated twice among the 45 post-ARSI samples.

To investigate the role of noncoding mutations in ARSI resistance, we performed a genome-wide search for noncoding SNVs and indels that were (a) newly arising after ARSI and (b) absent in all ARSI-naive tumors. The second rule was applied to effectively filter out many noncoding mutations of probably unknown significance. No such noncoding mutation was identified recurrently among the 45 pairs.

**Figure 2:**
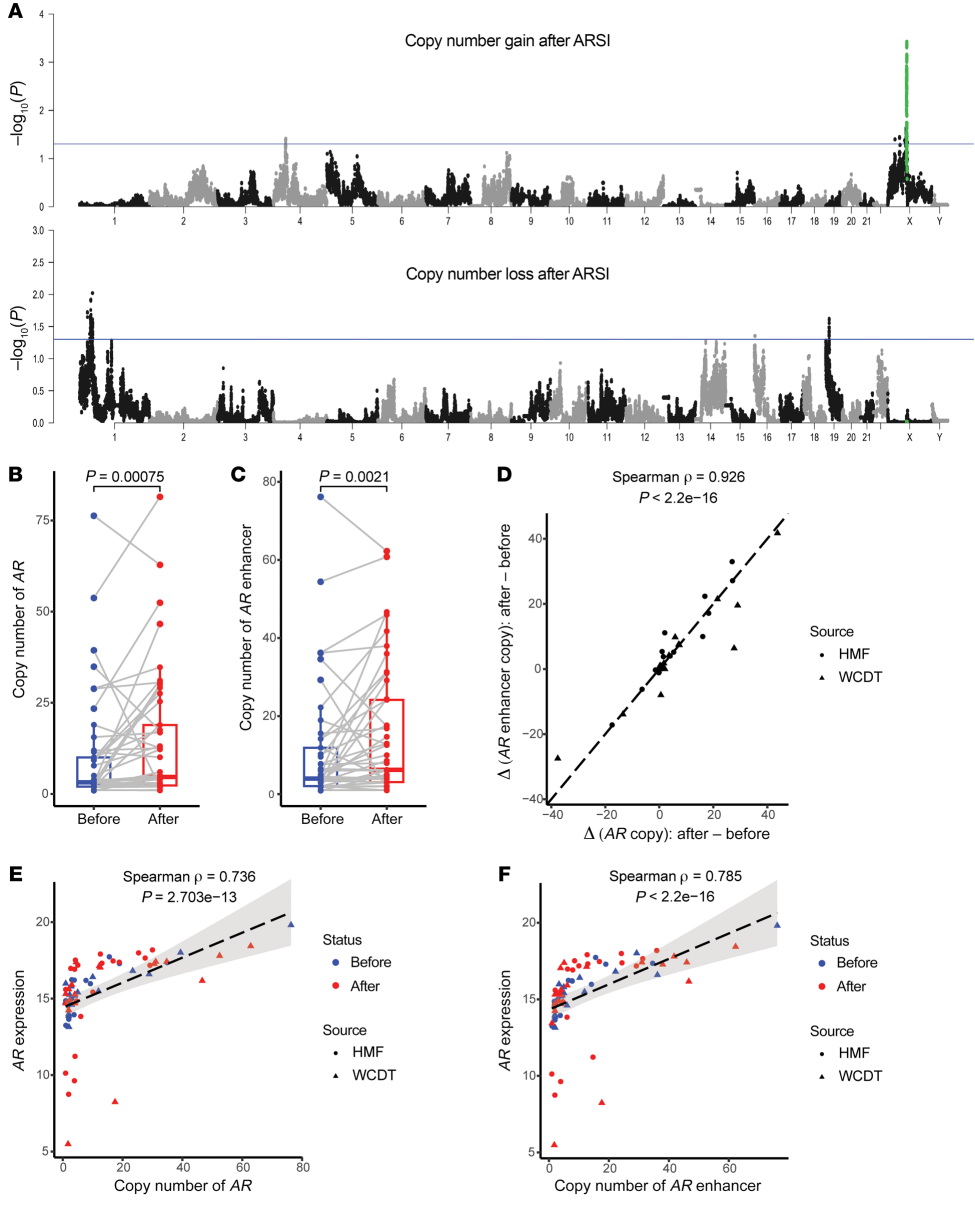
The *AR* locus is the major genetic substrate of converging evolution under ARSI-induced selective pressure. (**A**) Amplification of *AR* and its flanking sequences stood out as the predominant signal in genome-wide copy number analysis. The human genome was partitioned into 1 kb consecutive bins, and association tests were performed for each bin against the null hypotheses of (a) no pair gaining 1 or more copies after progression on ARSIs (top panel) and (b) no pair losing 1 or more copies thereof (bottom panel), respectively. *P* values were calculated using the paired Wilcoxon test for the 45 WGS pairs. *x* axis: chromosomal location with chromosomes numbered; *y* axis: –log_10_ (*P* value). Each dot represents an association test *P* value (–log_10_-transformed) for a given genomic bin, and 2 alternating colors (gray and black) were used to facilitate the visualization of genomic bins of consecutive chromosomes. The blue horizontal line in each panel indicates the threshold of nominal statistical significance (*P* < 0.05) to aid the visualization of potential hits. The *AR* locus (*AR* gene ±1 Mb flanking regions) is labeled in green. (**B** and **C**) mCRPC continues to acquire additional copies of *AR* and its upstream enhancer, reported by Quigley et al. (5), while developing ARSI resistance. *P* values were calculated using the paired Wilcoxon test (*n* = 45). (**D**) Copy number gains of *AR* and its upstream enhancer were highly correlated. (**E** and **F**) Higher *AR* and upstream enhancer copy numbers were correlated with higher *AR* mRNA levels.

Next, to systematically identify recurrent copy number alterations (CNAs) associated with ARSI resistance, we performed a genome-wide search by partitioning the human genome into consecutive 1 kb bins. For each bin, we tested the difference in copy number (calculated as the weighted average of segmented copy number estimates overlapping each bin) between pre- and post-ARSI paired samples. This analysis identified amplification of the *AR* locus (*AR* and its flanking regions) as the predominant post-ARSI change (Figure 2A). Notably, both *AR* and its upstream enhancer reported by Quigley et al. and others (5–7) gained additional copies after ARSI (Figure 2, B–D, and Supplemental Figure 4), accompanied by increased *AR* mRNA (Spearman ρ = 0.736, *P* = 2.703 × 10^–13^ and Spearman ρ = 0.785, *P* < 2.2 × 10^-16^ for *AR* and its upstream enhancer, respectively; Figure 2, E and F). Beyond the *AR* locus, we observed copy number gains in chromosome 4 (chr4: 43609001-43964000) and copy number losses in chromosomes 1 and 19 (chr1: 46038001-46044000, involving *PIK3R3* and chr19: 12186001-12192000, involving *ZNF136*; Figure 2A), achieving nominal statistical significance (*P* < 0.05). Given the multiple testing burden and lack of a strong rationale for a priori testing of these CNAs (unlike those involving the *AR* locus), further research is needed to evaluate the statistical evidence of these CNAs as related to ARSI resistance.

To investigate the potential differential effect of *AR* mutations and *AR* locus amplifications on ARSI resistance, we compared the duration of response (DOR) to ARSIs (abiraterone or enzalutamide) with respect to *AR* alterations. We found that *AR* mutation status (mutated versus nonmutated) was not associated with the DOR to first-line ARSI for mCRPC, in either post-ARSI or pre-ARSI tumors (Supplemental Figure 5, A and D). Similarly, *AR* gene/enhancer amplification status (defined as amplified if the *AR* copy was ≥4 or the upstream *AR* enhancer copy was ≥4, nonamplified if the *AR* copy was <4 and the upstream *AR* enhancer copy was <4) was not associated with DOR to first-line ARSI (Supplemental Figure 5, B and E). As *AR* mutations and *AR* gene/enhancer amplifications are not mutually exclusive (in fact, they tend to co-occur; Supplemental Figure 1), we further stratified our analyses into 4 groups (i.e., WT, mutated only, amplified only, and amplified and mutated). We again did not find significant differences in the DOR to first-line ARSI (Supplemental Figure 5, C and F); specifically, there was no difference between the “mutated-only” and “amplified-only” groups. Altogether, the similar responses to ARSIs in *AR*-mutated and *AR*-amplified tumors were consistent with the notion that these genetic alterations converge on augmenting AR function to compensate for AR inhibition (20).

Tandem duplication (TD) and extrachromosomal DNA (ecDNA) are 2 well-recognized mechanisms causing copy number gains of the *AR* locus (5, 28). Recently, the role of ecDNA in carcinogenesis and treatment resistance has been actively studied in multiple cancer types (29, 30), including our own work in mCRPC (31). In this study, we focused our analysis on TDs. We found that 36 (65.5%) and 37 (67.3%) of the 55 *AR* gene/enhancer-amplified (defined as above) tumors had TDs involving *AR* and its upstream enhancer, respectively (Supplemental Table 4). Among the 55 *AR* gene/enhancer-amplified tumors, we found a higher *AR* copy number in those without underlying TDs (Supplemental Figure 6A) and a similar trend for the copy number of the upstream *AR* enhancer (Supplemental Figure 6B). ecDNA is known to generate particularly high-level amplifications (29), and our finding suggests that ecDNA may be the mechanism driving *AR* gene/enhancer amplifications in the absence of TDs.

Finally, we examined the post-ARSI transcriptional changes of AR-V7 (32) and the androgen biosynthesis pathway (33), given their known importance in hormonal therapy resistance. AR-V7 isoform expression was quantified as the proportion of RNA-Seq reads supporting this specific splice variant relative to all reads mapped to any *AR* transcript. We compared all 71 RNA-Seq samples (31 pre-ARSI and 40 post-ARSI) and did not find a significant difference in the fraction of either AR-V7 (Supplemental Figure 7A) or full-length AR (AR-FL, Supplemental Figure 7B). We then evaluated the 31 paired RNA-Seq samples and did not find a significant difference either (Supplemental Figure 7, C and D). However, in 1 pair (HMF-019), we observed a dramatic increase in the AR-V7 fraction (0.1% → 60.7%) after ARSI, with a concurrent decrease in the AR-FL fraction (75.5% → 14.8%); in another pair (HMF-006), we found a moderate but probably meaningful increase in both AR-V7 (4.3% → 12.7%) and AR-FL fractions (13.5% → 20.3%) fractions after ARSI. To assess the transcriptional activity of the androgen biosynthesis pathway, we conducted single-sample gene set enrichment analysis (ssGSEA) (34) for the Reactome pathway (35) “androgen biosynthesis” and performed comparisons using all and paired RNA-Seq samples, respectively. We identified no significant changes (Supplemental Figure 8, A and B). For paired samples, there was a diverging change in androgen biosynthesis activity (Supplemental Figure 8, B and C), although all 3 pairs transforming into AR-indifferent phenotypes (see below) showed numerically lower activity (Supplemental Figure 8D).

Collectively, our systematic comparisons of paired mCRPC genomes highlighted the *AR* locus as the primary genetic substrate of convergent evolution to augment AR function under ARSI-induced selective pressure.

### A putative enhancer downstream of AR is amplified after ARSI treatment.

**Figure 3:**
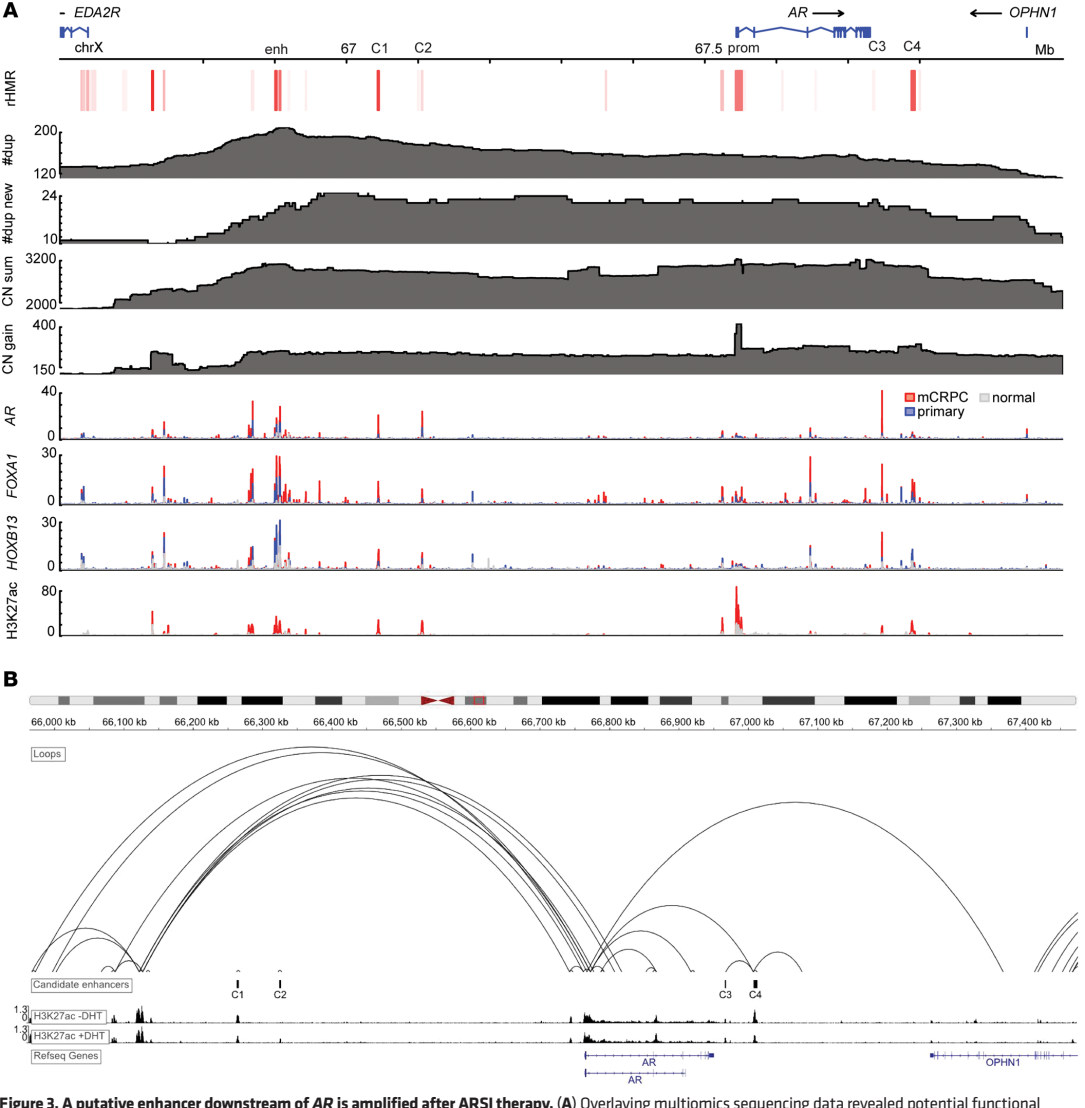
A putative enhancer downstream of *AR* is amplified after ARSI therapy. (**A**) Overlaying multiomics sequencing data revealed potential functional elements (C1–C4) flanking *AR* associated with ARSI resistance. enh, the known enhancer upstream of *AR* reported by Quigley et al. (5); prom, AR promoter; C1–C4, candidate functional elements (C1: chrX: 67043000-67046000; C2: chrX: 67104300-67106900; C3: chrX: 67746500-67748100; C4: chrX: 67787800-67793300; hg38); rHMR, recurrent hypomethylated regions in 100 mCRPC biopsies identified using WGBS reported by Zhao et al. (36) (redness indicates the frequency of recurrence); #dup, total number of TD events overlapping each base pair, identified by WGS of 201 mCRPC biopsies (*n* = 156 WCDT and *n* = 45 HMF samples); #dup new, total number of TD events overlapping each base pair, newly emerging after progression of disease on ARSIs, identified by WGS of 45 paired mCRPC biopsies; CN sum, copy number per base pair summed over the 201 mCRPC biopsies; CN gain, copy number gain (after ARSI – before ARSI) summed over the 45 paired mCRPC biopsies. Bottom 4 tracks show ChIP-Seq data for AR, FOXA1, HOXB13, and H3K27ac generated in normal prostate epithelium, primary PCa, and mCRPC, respectively (37). (**B**) HiChIP of H3K27ac in LNCaP cells (data were generated by Giambartolomei et al., ref. 40) demonstrates evidence of chromatin looping between C4 and the *AR* promoter.

Given the crucial role of the AR in PCa and ARSI resistance, we examined the amplified sequences flanking *AR* in detail, aiming to identify functional elements in ARSI-resistant tumors. We leveraged the multiomics data previously generated by our group (5, 36) and Pomerantz et al. (37) from mCRPC biopsies and patient-derived xenografts (PDXs) and attempted to identify putative functional DNA elements under ARSI-induced selective pressure supported by multiple lines of orthogonal genomic and epigenomic evidence. Specifically, we searched for DNA sequences that were (a) amplified in mCRPC and further amplified after ARSI (genetic evidence supporting relationship with ARSI resistance); (b) recurrently hypomethylated in mCRPC (36) (epigenetic evidence supporting potential active regulation of transcription); and (c) harboring ChIP-Seq peaks of key transcription factors (TFs) in mCRPC (AR, FOXA1, and HOXB13) and the active enhancer mark H3K27ac (additional evidence supporting potential functional significance). Although genomic regions related to active transcription are not always hypomethylated (38), we elected to focus on those recurrently hypomethylated regions (rHMRs) in this analysis because they have been shown to characterize mCRPC in our previous whole-genome bisulfite sequencing (WGBS) analysis (36). This integrated analysis successfully identified strong signals in the *AR* promoter and its upstream enhancer (5–7), both serving as positive controls, and highlighted 4 additional candidate regions (C1–C4 in Figure 3A).

To further assess the functional effect of these 4 candidate regions, we examined the publicly available dataset of chromosome conformation capture (3C) coupled with immunoprecipitation (HiChIP) of H3K27ac, a histone mark of active enhancers and promoters (39), generated from the LNCaP PCa cell line (40). We observed evidence for chromatin looping between C4 (chrX:67787800-67793300), but not C1–C3, and the *AR* promoter (Figure 3B). The copy number of C4 significantly increased (*P* = 0.0013; Supplemental Figure 9) after ARSI therapy, similar to the copy number gains involving *AR* (Figure 2B) and its upstream enhancer (Figure 2C). Furthermore, we found that 18 of the 42 (42.9%) pre-ARSI tumors and 22 of the 45 (48.9%) post-ARSI tumors harbored TDs involving C4, respectively, and that 10 of the 45 (22.2%) post-ARSI tumors gained new TDs involving C4. Unlike C1–C3, C4 seemed to lack a prominent AR ChIP-Seq signal but maintained FOXA1 binding in mCRPC (Figure 3A). To explore additional TFs that may interact with the DNA sequences within C4, we performed a GIGGLE enrichment analysis (41) using all ChIP-Seq profiles available via cistromeDB (42, 43). In line with ChIP-Seq data from patient samples (Figure 3A), the AR was found to be the top TF binding to C1–C3 but not to C4 (Supplemental Figure 10). To nominate candidate TFs binding to C4 in an unbiased manner, we leveraged the recently published assay for transposase-accessible chromatin sequencing (ATAC-Seq) data from human CRPC organoids (17). We identified 3 and 4 ATAC-Seq peaks within C1–C3 and C4, respectively, performed TF motif enrichment analysis for these peaks (44), and observed distinct TF binding profiles (Supplemental Figure 11). Whereas steroid receptors were predicted to bind to C1–C3, a very different set of TFs were predicted to bind to C4, among which we found *FOXA1* to be significantly coexpressed with *AR* (Supplemental Figure 12). To further examine FOXA1 binding in C4, we queried the ReMap database (45) and observed evidence of FOXA1 binding in multiple PCa cell lines, primary PCa, and mCRPC (Supplemental Figure 13). Given the reported involvement of FOXA2 in lineage plasticity and NE PCa (46) but the lack of FOXA2 signal in ReMap (45), we searched the ChIP Atlas database (47) and found 2 ChIP-Seq peaks located in C4 reported by a recent study (48). Altogether, our multiomics analyses pinpoint a noncoding DNA element downstream of *AR* that is amplified in mCRPC and may mediate ARSI resistance by potentially interacting with TFs including FOXA1 and possibly FOXA2, calling for further research to understand the significance and mechanisms of these findings.

### Unpaired analysis reveals increased transcriptional heterogeneity in ARSI-resistant mCRPC.

**Figure 4:**
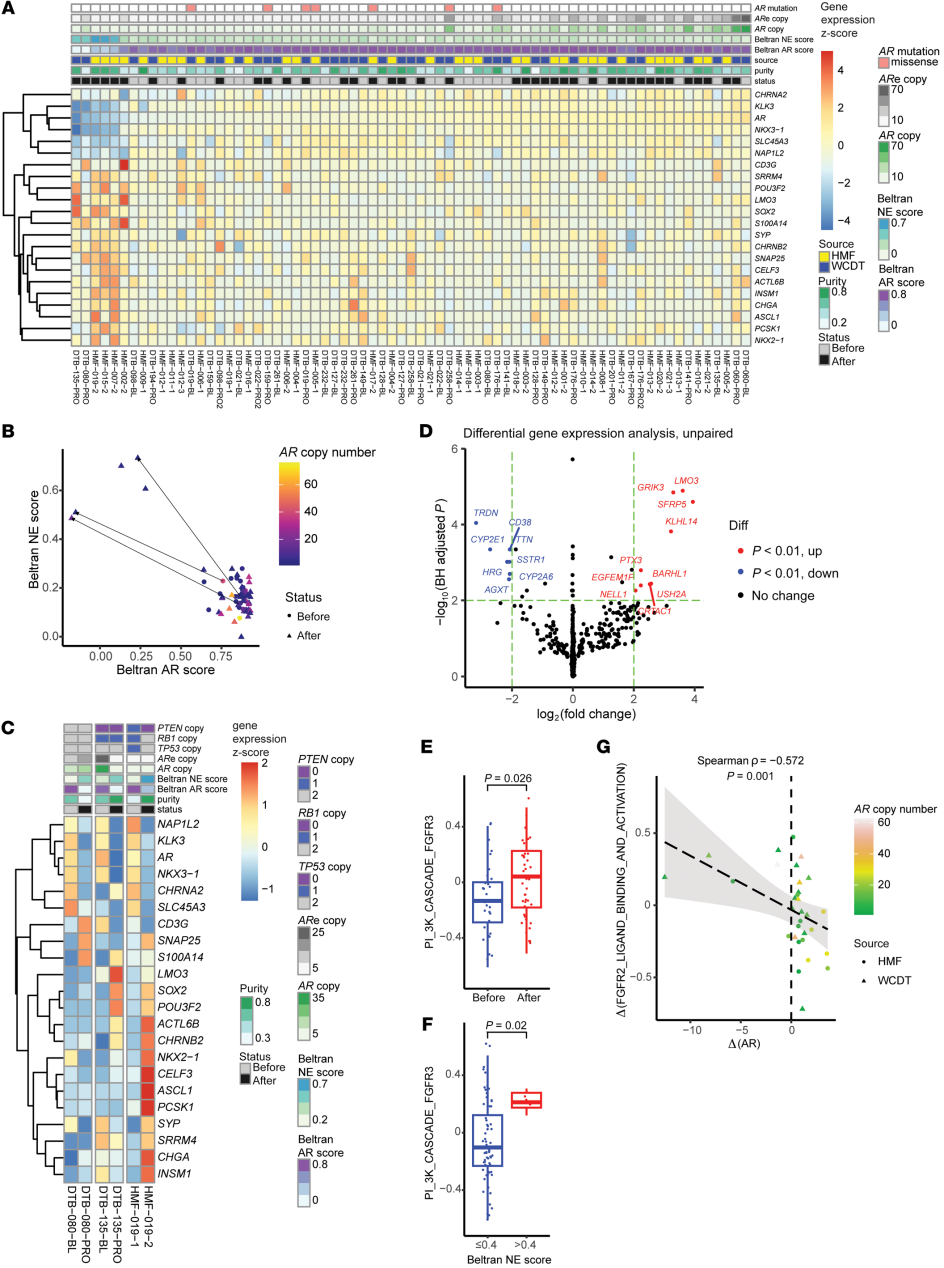
Transcriptomics analyses comparing mCRPC tumors before and after ARSIs. (**A**) Heatmap of the 22 genes used to subtype mCRPC by Labrecque et al. (11), sorted by *AR* expression. Both extremes of the *AR* expression spectrum were enriched with ARSI-resistant tumors, indicating diverging changes. ARe, upstream *AR* enhancer reported by Quigley et al. (5). (**B**) Scatter plot of AR and NE scores calculated per Beltran et al. (9). Directed line segments indicate the 3 pairs showing a clear post-ARSI phenotypic switch, 2 of which (the 2 WCDT pairs) were also reported by Westbrook et al. (13). All 5 NE-high samples are post-ARSI samples without a high *AR* copy number. (**C**) Focused heatmap of the 3 phenotypic converters in **B** highlights the transcriptional heterogeneity within this group. (**D**) Unpaired DGE analysis identified relevant genes involved in ARSI-resistant tumors, including *LMO3* (a NE TF and 1 of the 22 Labrecque genes) and the Wnt signaling regulator *SFRP5* (Wald test, DESeq2). diff, differential. (**E**) Unpaired DGE analysis of Reactome pathways highlighted that FGFR pathways were among the most upregulated in ARSI-resistant mCRPC (Wilcoxon test). (**F**) FGFR3 pathway activity was higher in mCRPC tumors with a high NE score (>0.4 as defined by Beltran et al. [ref. 9]; Wilcoxon test) (**G**) Changes in the FGFR2 pathway and *AR* expression were anticorrelated.

Despite the recurrent genetic alterations converging on augmenting AR function, mCRPC is well known to exhibit treatment-emergent transcriptional heterogeneity and lineage plasticity, the underlying mechanisms of which are only partially understood (9–11, 49). To assess the phenotypic evolution of mCRPC being treated with ARSIs, we first analyzed RNA-Seq data focusing on genes used to define molecular subtypes of mCRPC (9, 11). After correcting for the sample source (Supplemental Figure 14), we found that *AR* was among the top 50 most variably expressed genes (ranking 49/20810). Notably, tumors exhibiting both the highest and lowest levels of *AR* expression were predominantly those having progressed on ARSIs (Figure 4A). Indeed, *AR* expression became more variable after ARSI therapy (*P* = 1.52 × 10^–4^, *F* test). Furthermore, all 22 genes used for molecular subtyping of mCRPC by Labrecque et al. (11) showed increased variability of post-ARSI expression (Supplemental Figure 15). Tumors with higher *AR* expression tended to have more copies of *AR* and its upstream enhancer (Figure 2, E and F, and Figure 4A), whereas tumors with lower *AR* expression tended to lack such amplifications. Corroborating prior reports (13, 14), we used the Beltran AR/NE scoring system (9) and observed tumors that transitioned from a high AR/low NE state to a low AR/high NE state (3 of 31 RNA-Seq pairs; Figure 4, B and C). Among these 3 pairs, 1 (HMF-019) transitioned to a clear NE phenotype, supported by the highly expressed NE TFs (e.g., *NKX2-1*, *ASCL1*, and *INSM1*) and markers (e.g., *CHGA*; Figure 4C), while the other 2 (DTB-080 and DTB-135) had a lower NE score with correspondingly less prominent expression of NE-related genes after ARSI therapy (11, 50).

Inspired by the recent progress in understanding mCRPC heterogeneity using advanced sequencing technologies such as ATAC-Seq, we similarly calculated phenotypic scores for each tumor using the key TFs defining the 4 molecular subtypes of mCRPC reported by Tang et al. (17): AR-dependent (CRPC-AR), NE (CRPC-NEPC), Wnt-dependent (CRPC-Wnt), and stem cell-like (CRPC-SCL). Despite a lack of consistent changes achieving statistical significance when comparing the 31 RNA-Seq pairs for each subtype score, we observed tumors that had large post-ARSI numerical changes in these scores, indicating treatment-emergent transcriptional rewiring (Supplemental Figure 16, A–D). Post-ARSI changes of these subtype scores were often uncorrelated (Supplemental Figure 16E), indicating that they tend to measure orthogonal features of the tumor transcriptional profile. To identify pairs with exceptional state transitions after ARSI, we performed a principal component analysis using the changes in the 4 subtype scores and identified 4 outliers (Supplemental Figure 16F), 3 of which (DTB-080-PRO, DTB-135-PRO, and HMF-019-2) were also identified as phenotypic converters using the Beltran AR/NE scoring system (Figure 4, B and C). Concordant with the transcriptional heterogeneity assessed using the Labrecque genes (11) for these 3 pairs (Figure 4C), we observed variable changes in Wnt, NEPC, and SCL scores despite a more consistent decrease in the AR score (Supplemental Figure 16, G–I). The fourth outlier, HMF-012-3 (Supplemental Figure 16F), maintained a relatively high AR post-ARSI score (Supplemental Figure 16, G-I) but lower Wnt, NEPC, and SCL scores (Supplemental Figure 16, J–L), suggesting increased AR addiction or transition to a resistance phenotype not yet well captured by these scores. These findings also agree with the notion that the transcriptional phenotypes of mCRPC tumors, particularly those that have been treated with multiple hormonal therapy agents, represent a continuum rather than discrete states (11).

To further determine the transcriptional features of ARSI-resistant tumors, we conducted a genome-wide analysis to identify differentially expressed genes by comparing all post-ARSI (*n* = 40) versus pre-ARSI (*n* = 31) samples (unpaired analysis). *LMO3* was found to be the most significantly upregulated gene after ARSI therapy (Figure 4D). *LMO3* is a TF that regulates NE differentiation and 1 of the 22 Labrecque genes used to molecularly subtype mCRPC (11); consistently, *LMO3* expression increased in the 3 tumors, gaining a high Beltran NE score following ARSI (Figure 4, B and C). A query of ReMap (45) showed ChIP-Seq signals of FOXA1 in the promoter of *LMO3* in mCRPC (51) that were distinct from those in primary PCa (52), indicating the rewiring of FOXA1 cistrome as a potential upstream event (Supplemental Figure 17). *SFRP5*, encoding a soluble regulator of Wnt signaling (53), was also significantly upregulated after ARSI (Figure 4D), highlighting the known importance of Wnt signaling in ARSI resistance (2, 17). To search for biological pathways associated with ARSI resistance, we conducted unpaired ssGSEA (34) and identified FGFR pathways among the most significantly upregulated, most prominently FGFR3 (Figure 4E and Supplemental Figure 18A). Notably, all 5 tumors with a high NE score were ARSI-resistant (Figure 4B) and had a high transcriptional activity of the FGFR3 pathway (Figure 4F). Among all Reactome pathways, change in the FGFR2 pathway was the most anticorrelated with change in *AR* expression (Figure 4G and Supplemental Figure 18B). These findings are consistent with prior studies reporting that FGFR signaling supports lineage plasticity and AR-independent PCa (12, 54, 55).

### Paired analysis identifies transcriptional downregulation of SSTR1 in ARSI-resistant mCRPC.

**Figure 5:**
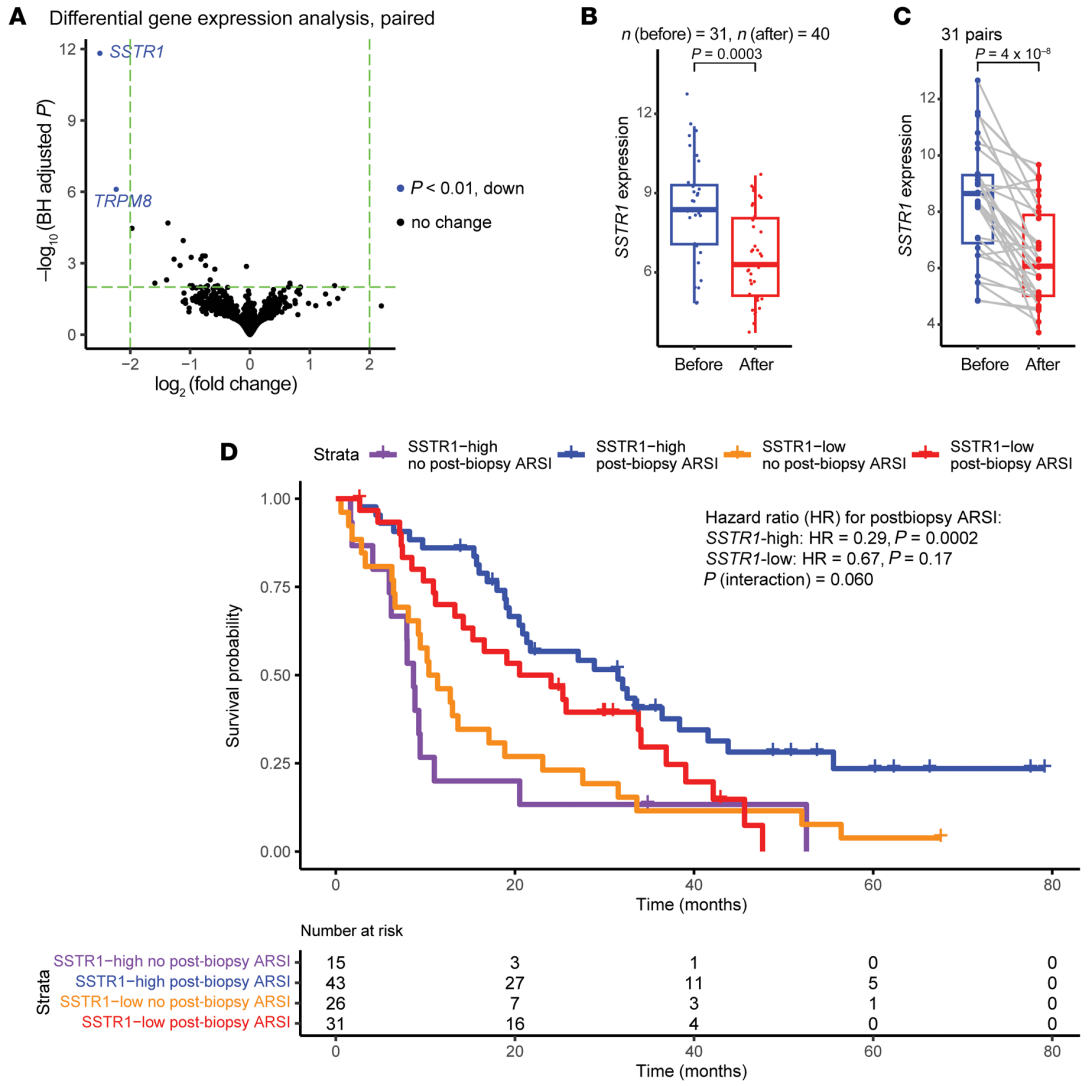
Decreased *SSTR1* mRNA in ARSI-resistant mCRPC. (**A**) Paired DGE analysis identifies *SSTR1* as the most significantly altered gene after ARSI therapy (Wald test, DESeq2). BH, Benjamini-Hochberg procedure. (**B**) *SSTR1* mRNA decreased, while mCRPC developed ARSI resistance in an unpaired analysis using all 71 RNA-Seq samples (Wilcoxon test). (**C**) *SSTR1* downregulation was consistently observed after ARSI across 31 paired samples (paired Wilcoxon test). (**D**) High *SSTR1* expression was associated with survival benefit in 115 WCDT patients who received ARSIs following the biopsy (Wald test).

Paired analysis of metastatic biopsies from the same patients before and after treatment offers a unique advantage in identifying resistance mechanisms conserved among patients. We performed paired differential gene expression (DGE) analysis to focus on post-ARSI changes after accounting for patient-unique factors. Our genome-wide analysis identified *SSTR1* as the most significant hit; its expression consistently decreased in most patients after disease progression on ARSIs (Figure 5, A–C). *SSTR1* encodes somatostatin receptor 1, a G protein–coupled receptor (GPCR) on the plasma membrane that mediates antiproliferative, antimigratory, and antisecretory effects when activated by its endogenous ligand, somatostatin (56). Somatostatin is a peptide that functions as a neurotransmitter in the CNS and as a hormone regulating the endocrine system by binding to somatostatin receptors 1–5 (*SSTR1*–*SSTR5*) (56). Given their antitumor effects, somatostatin analogs such as octreotide and lanreotide (both are potent SSTR2 agonists but notably spare SSTR1) have been approved to treat gastroenteropancreatic NE tumors and carcinoid syndrome (57, 58). Among different cancer types in TCGA, *SSTR1* mRNA in primary PCa is higher than all the other cancer types except glioma (26, 59) (Supplemental Figure 19). Using a recently published single-cell RNA-Seq dataset of mCRPC biopsies (60), we found that *SSTR1* expression was predominantly restricted to PCa cells and absent in the tumor immune microenvironment (60, 61) (Supplemental Figure 20).

Our analysis revealed no apparent correlation between the *SSTR1* copy number and its mRNA level (Supplemental Figure 21A). We examined the associations between *SSTR1* expression and known ARSI resistance mechanisms including *AR* amplification and lineage plasticity. *SSTR1* expression was not associated with the *AR* copy number, the upstream *AR* enhancer copy number, *AR* expression, the AR score, or the NE score (Supplemental Figure 21, B–F).

In the 115 Stand Up To Cancer/Prostate Cancer Foundation (SU2C/PCF) West Coast Dream Team (WCDT) patients (retrospectively ascertained) with both survival and RNA-Seq data available, we found that lower *SSTR1* mRNA expression was associated with reduced benefit from ARSIs following biopsy (interaction *P* = 0.0598, Figure 5D) and worse overall survival (Supplemental Figure 22A). Among ARSI-naive patients (*n* = 54), no differential benefit of ARSIs was observed with respect to *SSTR1* expression (Supplemental Figure 22B), as opposed to ARSI-experienced patients (*n* = 61, Supplemental Figure 22C). This observation is consistent with a correlation between *SSTR1* downregulation and acquired ARSI resistance.

### SSTR1 expression is linked to AR signaling.

To understand the biological mechanisms underlying the association between *SSTR1* downregulation and ARSI resistance, we expanded our analysis to include all samples from the WCDT cohort with RNA-Seq data available. We assigned 181 mCRPC tumors to *SSTR1*-high or *SSTR1*-low groups using the median *SSTR1* mRNA level as the cutoff and then compared their transcriptomes. Several steroidogenesis genes stood out as being significantly upregulated in *SSTR1*-low tumors and included *CYP17A1*, *STAR*, and *CYP21A2* (Supplemental Figure 23A), which was attributable to 3 outlier tumors (Supplemental Figure 23, B and C). When comparing these 3 tumors with the rest in the dataset, ssGSEA revealed that steroidogenesis pathways represented 3 of the 5 pathways most significantly upregulated (Supplemental Figure 23D). These results revealed excessive synthesis of steroid hormones in some *SSTR1*-low, ARSI-resistant tumors.

**Figure 6:**
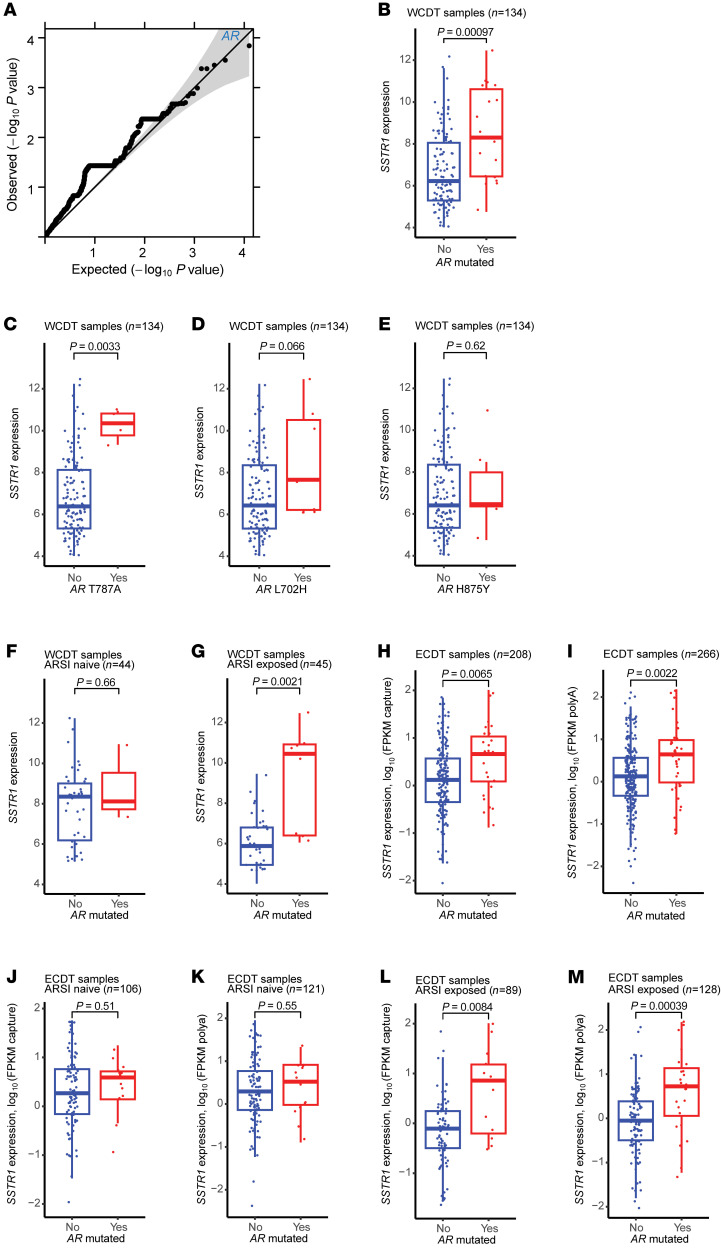
*AR* mutations are associated with higher *SSTR1* mRNA in ARSI-exposed mCRPC. (**A**) Quantile-quantile plot of *P* values (*t* test as implemented in the linear regression model) shows *AR* to be the gene most significantly associated with *SSTR1* mRNA expression. (**B**) *AR*-mutated mCRPC had higher *SSTR1* expression in the WCDT. (**C**–**E**) Single-mutation analysis in WCDT samples demonstrated T878A to be the main contributor to the gene-level association, with L702H and H875Y showing trends in the same direction. (**F** and **G**) *AR*-mutated status predicted higher *SSTR1* expression only in ARSI-exposed tumors in the WCDT dataset. (**H** and **I**) The positive association between *AR* mutation status and *SSTR1* mRNA was replicated in the ECDT dataset, where analyses were stratified by the RNA-Seq method (capture vs. polyA). (**J**–**M**) Similarly, in the ECDT dataset, *AR*-mutated status predicted higher *SSTR1* expression in ARSI-exposed, but not ARSI-naive, mCRPC. All *P* values were calculated using the Wilcoxon test for **B**–**M**.

To investigate the effect of DNA mutations on *SSTR1* expression in mCRPC (as it is not trivially explained by *SSTR1* copy number), we conducted a genome-wide association analysis using the 134 WCDT mCRPC samples with both WGS and matched RNA-Seq data available. We tested the association between *SSTR1* mRNA and the burden of functional protein-coding mutations in each gene, using a linear regression model adjusting for tumor purity (62). Among the 6,277 evaluable genes (each with at least 1 functional protein-coding mutation), *AR* demonstrated the strongest association with *SSTR1* mRNA (*P* = 1.44 × 10^–4^; Figure 6A). *AR*-mutated tumors had higher *SSTR1* expression (Figure 6B), independent of the *AR* copy number (Supplemental Figure 24A). To assess the effect of individual *AR* mutations, we performed single-variant analysis and found that T878A and, to a lesser extent, L702H were associated with *SSTR1* mRNA (Figure 6, C and D), whereas H875Y was not (Figure 6E). Interestingly, the positive association between *AR* mutations and *SSTR1* expression was observed only in ARSI-exposed, but not ARSI-naive, tumors (Figure 6, F and G). Further analysis using multiple linear regression confirmed that both ARSI exposure status (exposed vs. naive) and *AR* mutation status (mutated vs. WT) independently predicted *SSTR1* expression (β = –1.62, *P* = 5.88 × 10^–5^ and β = 2.53, *P* = 3.84 × 10^–5^, respectively), with a trend toward significant statistical interaction (*P* = 0.067), whereas tumor purity had no effect (*P* = 0.82). To validate our findings in an independent cohort, we evaluated the SU2C/PCF East Coast Dream Team (ECDT) dataset (63). Similarly, we found that *AR* mutations were significantly associated with high *SSTR1* expression (Figure 6, H and I), with L702H and H875Y showing statistical significance in single variant analysis (Supplemental Figure 24, B–I). Further, *AR* mutation status predicted *SSTR1* expression only in ARSI-exposed, but not ARSI-naive, tumors in the ECDT dataset (Figure 6, J–M). Collectively, these data suggest that *SSTR1* expression is linked to AR signaling in mCRPC.

### SSTR1 upregulation is associated with the response to high-dose testosterone.

**Figure 7:**
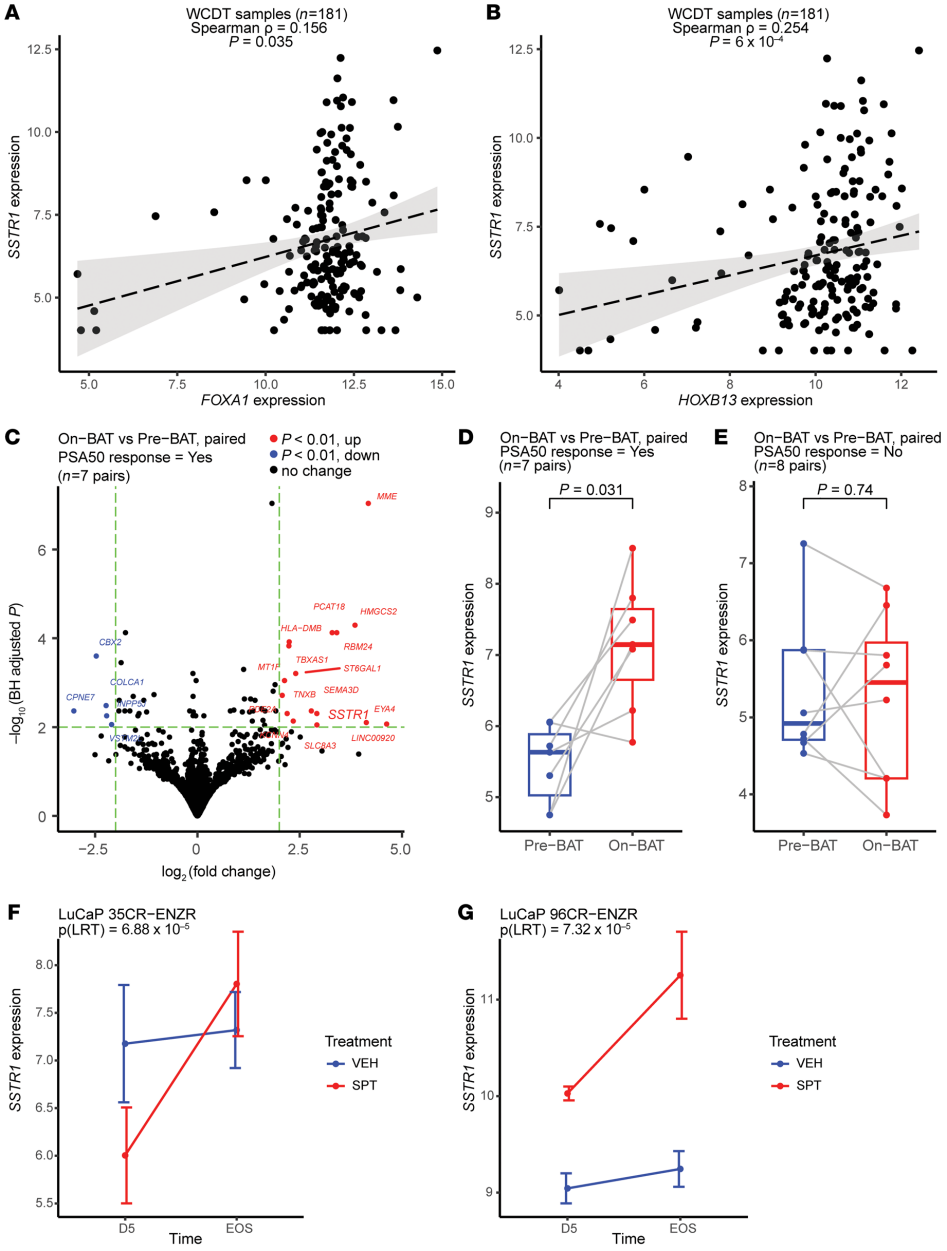
*SSTR1* is potentially regulated by the AR/FOXA1/HOXB13 transcription machinery, and its upregulation is associated with tumor response to high-dose testosterone. (**A** and **B**) *FOXA1* and *HOXB13* were each coexpressed with *SSTR1* in the WCDT cohort. (**C** and **D**) *SSTR1* is one of the most significantly upregulated genes in mCRPC tumors that responded to BAT from the COMBAT-CRPC trial (data from Sena et al.; ref. 67). (**E**) Conversely, no change in *SSTR1* expression was observed in tumors without a PSA50 response. (**F** and **G**) Echoing findings in patients, SPT suppressed mCRPC tumor growth in 2 enzalutamide-resistant PDX models, LuCaP 35CR-ENZR and LuCaP 96CR-ENZR; in both models, SSTR1 expression was upregulated after SPT. D5, day 5 after SPT; EOS, end of study, as reported by Lam et al. (68); VEH, vehicle; LRT, likelihood ratio test. *P* values in **C** were calculated using the Wald test (DESeq2); *P* values in **D** and **E** were calculated using the paired Wilcoxon test.

Motivated by our findings of *SSTR1* downregulation in ARSI-resistant PCa and the association between *SSTR1* expression and *AR* mutations, we further investigated whether the AR and its cooperating TFs could affect *SSTR1* expression. Although not recognized as a classic AR target gene, an examination of cistromeDB revealed AR and FOXA1 ChIP-Seq peaks near the *SSTR1* locus in both LNCaP and VCaP cells and HOXB13 ChIP-Seq peaks in LNCaP cells (42, 43). In ReMap (45), we observed ChIP-Seq signals of AR, FOXA1, and HOXB13 in mCRPC PDXs (51) (Supplemental Figure 25). Furthermore, although *AR* and *SSTR1* mRNA levels were uncorrelated (Supplemental Figure 21D and Supplemental Figure 26, A and B), *FOXA1* and *HOXB13* were each moderately coexpressed with *SSTR1* in both WCDT and ECDT (Figure 7, A and B, and Supplemental Figure 26, C–F).

Downregulation of *SSTR1* in patients with PCa whose disease is progressing while on ARSIs is consistent with the known antitumor effect of somatostatin (56) and somatostatin analogs (57, 58) observed in other cancer types (e.g., gastrointestinal and pancreatic NE tumors). We thus hypothesized that augmenting *SSTR1* expression or SSTR1 signaling (e.g., using an agonist) may suppress tumor growth and serve as a therapeutic avenue for ARSI-resistant PCa. Since *SSTR1* is downregulated after ARSI, we examined the reciprocal relationship between the AR/FOXA1/HOXB13 transcription machinery (64, 65) and *SSTR1* expression, specifically the effect of testosterone (as opposed to ADT and ARSIs) on *SSTR1* expression and tumor growth. In this context, bipolar androgen therapy (BAT), the rapid cycling of supraphysiologic androgen and androgen ablation, has improved outcomes in some patients with mCRPC, yet the biomarkers that reliably predict responses remain undefined (66). By analyzing the RNA-Seq data on paired metastatic biopsies obtained before and after 3 cycles of BAT (administered concurrently with the anti–PD-1 antibody nivolumab) from patients with mCRPC enrolled in the Concurrent Administration of BAT–CRPC (COMBAT-CRPC) trial (67), we identified *SSTR1* as one of the most significantly upregulated genes after BAT in the 7 patients whose prostate-specific antigen (PSA) levels decreased by 50% or more (i.e., PSA50 response) after treatment (Figure 7, C and D). Conversely, in the 8 patients without a PSA50 response, *SSTR1* expression remained unchanged (Figure 7E). Furthermore, we analyzed the RNA-Seq data from a preclinical study of supraphysiological testosterone (SPT) in mCRPC PDXs (68). SPT suppressed tumor growth and prolonged the survival of host mice in 2 enzalutamide-resistant models, LuCaP 35CR-ENZR and LuCaP 96CR-ENZR (68), and *SSTR1* expression increased after SPT in both models (Figure 7, F and G). These findings suggest that upregulated *SSTR1* expression was associated with the tumor-suppressive effect of high-dose testosterone observed in some patients and preclinical models of mCRPC.

### SSTR1 is a promising drug target in mCRPC.

Intrigued by the above findings of *SSTR1*, we conducted experiments in PCa cells to further investigate the relationship between SSTR1 and AR signaling. We selected the enzalutamide-resistant 22Rv1 cells to represent ARSI-resistant mCRPC; 22Rv1 cells also have higher *SSTR1* expression compared with several other PCa cell lines (69). We found that dihydrotestosterone (DHT) treatment did not seem to change *SSTR1* mRNA expression at 48 hours (Supplemental Figure 27A). Next, we performed *SSTR1* stable knockdown (using clustered, regularly interspaced palindromic repeats interference [CRISPRi]) and stable overexpression assays in 22Rv1 cells (Supplemental Figure 27, B and C) and found no obvious effect on *AR* mRNA of either silencing or overexpressing *SSTR1* (Supplemental Figure 27D). Consistent with our hypothesis, knockdown of *SSTR1* significantly augmented 22Rv1 cell proliferation (Supplemental Figure 27E), while inducing overexpression of *SSTR1* had the opposite effect (Supplemental Figure 27F). In line with similar findings from a prior study (70), our results validate the antitumor effect of SSTR1 in mCRPC.

Last, we investigated the feasibility of targeting SSTR1 to overcome ARSI resistance. Somatostatin receptors are well-established drug targets in benign NE conditions and NE tumors, with multiple somatostatin analogs approved by the FDA (58, 71). Octreotide and lanreotide, both SSTR2 agonists but sparing SSTR1, were tested in nonmetastatic CRPC (nmCRPC) and mCRPC (motivated by their effects on lowering circulating insulin-like growth factors) and found to have no definitive clinical benefit (72–74). We identified pasireotide as the only FDA-approved drug (a pan-SSTR agonist for treating acromegaly and Cushing disease) with SSTR1 agonist activity (75, 76) and evaluated the potential of repurposing it to overcome ARSI resistance. We found that pasireotide significantly suppressed 22Rv1 cell proliferation in vitro (Supplemental Figure 27G), whereas cyclosomatostatin (an SSTR1 antagonist) did not appear to have a substantive effect (Supplemental Figure 27H). Altogether, our data suggest that SSTR1 is antiproliferative in mCRPC and amenable to pharmacological intervention, potentially through the repurposing of pasireotide.

## Discussion

Optimizing the clinical benefit of ARSIs is crucial to improving the care for patients with PCa and depends on a thorough understanding of resistance mechanisms. To systematically investigate the mechanisms of ARSI resistance, we conducted an integrated genomic and transcriptomics analysis of paired mCRPC biopsies obtained before the initiation of and after disease progression on abiraterone or enzalutamide. To maximize the discovery power, we combined samples from the SU2C-PCF WCDT and the HMF cohorts and report here, to our knowledge, the largest integrated sequencing study of paired mCRPC biopsies.

The 2 well-recognized mechanisms of ARSI resistance are genetic alterations augmenting AR signaling (2, 5–7, 19, 20) and phenotypic evolution into apparently AR-indifferent tumors (9–11, 13, 14). Our unbiased analyses of paired biopsies confirm both mechanisms and further emphasize the prominent role of genetic alterations involving *AR* and its neighboring regulatory elements to counteract ARSI-induced selective pressure (Figure 2A). We did not find definitive evidence for non-*AR* genetic alterations driving ARSI resistance, and current literature reports similar observations through analysis of serial tissue (18) or ctDNA (20) specimens. Together, these observations suggest 2 hypotheses that are not mutually exclusive and can be tested in future research. First, non-*AR* genetic drivers of ARSI resistance may have a high genetic heterogeneity and follow a “long tail” distribution (77), and larger sample sizes are required to observe individual events at sufficient frequency for robust statistical inference. Second, epigenetic mechanisms (e.g., changes in DNA methylation, histone modifications, chromatin accessibility, and genome organization) may play a key role in ARSI resistance, and epigenomic profiling techniques may facilitate the identification of these mechanisms (31, 50, 78–80).

We dissected the frequently amplified *AR* locus, the primary substrate of convergent evolution for PCa to counteract ARSIs. By integrating multiomics datasets, we nominated a putative downstream *AR* enhancer that potentially interacts with key TFs such as FOXA1 (Figure 3). This finding, together with the prevalent amplification of the upstream *AR* enhancer in mCRPC (5–7), suggests an instrumental role of noncoding DNA in AR biology and ARSI resistance. Both the upstream (5–7) and downstream *AR* enhancers tend to coamplify with *AR*, so statistical discernment of their phenotypic effects (e.g., on *AR* expression and AR cistrome) is challenging, and experiments are needed to interrogate their function individually and combinatorially. In this study, we focused our analyses on multiomics sequencing data generated from patient samples and patient-derived cell lines. Therefore, the epigenomic datasets analyzed are limited to WGBS and ChIP-Seq data made available from prior extensive efforts of our group and others (36, 37). Obtaining and integrating more datasets by profiling additional TFs and chromatin modifiers such as C/EBPβ (38) and the SWI/SNF complex (81) may yield new insights through updated analyses in the future.

Transcriptional reprogramming and lineage plasticity are widely observed in mCRPC and are increasingly recognized as a critical resistance mechanism adopted by PCa and other cancer types (2, 12, 49). Our analyses revealed excessively variable expression of lineage-informative genes (e.g., the 22 Labrecque genes for mCRPC subtyping) (11) after ARSI therapy (Supplemental Figure 15). Such phenotypic diversification is remarkable, given the prevalent genetic alterations that monotonously converge on augmenting AR signaling, indicating additional mechanisms (such as non-*AR* genetic alterations or epigenetic mechanisms discussed earlier) in play. Similar to prior studies (13, 14), we identified tumor pairs showing a clear phenotypic switch from an AR-driven to an apparently AR-indifferent form, measured either by the Beltran scoring system (9) or the 4-subtype classification scheme recently reported by Tang et al. (17). We also found increased activity of FGFR pathways after ARSI therapy, particularly in tumors gaining a NE phenotype , corroborating recent reports (12, 54, 55).

Paired analysis is uniquely powered to identify resistance mechanisms conserved among patients by accounting for the effect of patient-specific factors. We identified *SSTR1* as the most significant gene and found its expression decreased in most ARSI-resistant tumors (Figure 5, A–C). In line with the near-ubiquity of this observation in our dataset, *SSTR1* downregulation occurred after ARSI treatment across the full phenotypic spectrum captured by the Beltran (9) or Tang (17) method. *SSTR1* expression is high in primary, untreated PCa and appears to be restricted to cancer cells (26, 59, 60). Our findings suggest that *SSTR1* expression may be a candidate predictive biomarker for response to second-line ARSIs (i.e., Figure 5D and Supplemental Figure 22, B and C) following disease progression on the first ARSI (82). With the increasing use of ARSIs upfront to treat PCa, whether patients should be rechallenged with a second ARSI at disease progression has become an important clinical question. In this clinical space with growing patient needs, a second ARSI may be preferred prior to cytotoxic drugs such as chemotherapy and radioligand therapy, given the ARSI’s favorable side-effect profile. However, its efficacy is hard to predict and can be highly variable (82, 83), as there is currently no predictive biomarker to guide this important clinical decision–making process. We provide preliminary data to support tumor *SSTR1* expression as a promising candidate for such a biomarker. Larger studies, ideally using a randomized controlled design, are needed to further evaluate this finding.

Our analyses of multiple independent datasets revealed an intriguing link between SSTR1 and AR signaling in mCRPC. The presence of *AR* mutations was associated with higher *SSTR1* expression (Figure 6 and Supplemental Figure 24), leading to the hypothesis that tumors with high *SSTR1* expression may be more delicately dependent on AR signaling (indicated by the acquisition of *AR* mutations) and thus more susceptible to ARSIs. Furthermore, we found that the AR/FOXA1/HOXB13 transcription machinery potentially regulates *SSTR1* and that *SSTR1* upregulation was associated with a response to high-dose testosterone in mCRPC (Figure 7). Our experiments using 22Rv1 cells did not show a significant change in *SSTR1* mRNA expression after 48 hours of DHT treatment (Supplemental Figure 27A); further studies with a longer time frame, more cell lines, and animal models may help increase our understanding of the dynamics of *SSTR1* expression as related to ARSIs or BAT/SPT in mCRPC. Although genetic modulation of *SSTR1* did not seem to affect *AR* mRNA (Supplemental Figure 7D), we observed a clear antiproliferative effect of SSTR1 in 22Rv1 cells (Supplemental Figure 7, E and F), consistent with the known SSTR1 biology and prior studies (56, 70). Importantly, we showed that pasireotide significantly suppressed 22Rv1 cell proliferation (Supplemental Figure 7G) and provided the proof of concept for targeting SSTR1, potentially by repurposing the FDA-approved drug pasireotide, to overcome ARSI resistance. These findings lay the groundwork for further research evaluating the therapeutic potential of enhancing SSTR1 function to improve patient outcomes.

Given its exceptionally strong statistical evidence and actionability potential, we focused our study on *SSTR1* as the most prominent hit from the paired DGE analysis. Interestingly, *TRPM8* (Figure 5A) was the only other strong finding based on the magnitude of change and statistical significance and has been studied in PCa by several research groups that reported different biological mechanisms. An initial study showed that TRPM8 was required for PCa survival (84), but subsequent studies found that both TRPM8 agonists (85) and antagonists (86) might have an antitumor effect in PCa, requiring further research to clarify. Furthermore, a recent study reported that *TRPM8* expression might promote antitumor innate immunity in PCa (87). Our finding of *TRPM8* downregulation in ARSI-resistant mCRPC complements these observations with relevant patient data and highlights the need for more research on this gene.

Our study has limitations. First, paired biopsies were obtained from patients with mCRPC, so we were not able to directly study the resistance mechanisms in HSPC. Second, the tumors studied were treated with either abiraterone or enzalutamide; newer ARSIs, such as apalutamide and darolutamide, were not studied because they were less commonly used at the time of patient recruitment. However, given the similar mechanisms of action, our findings may be cautiously extrapolated to these newer ARSIs. Third, despite being the largest genomic analysis of paired mCRPC biopsies to our knowledge, the number of patients was moderate, and a larger sample size may enable the discovery of additional features and mechanisms of ARSI resistance.

In summary, we report a genomic and transcriptomics analysis of paired mCRPC biopsies to investigate the mechanisms of ARSI resistance. We identified a putative downstream *AR* enhancer amplified in ARSI-resistant tumors. In addition, we discovered SSTR1 as a candidate biomarker to predict the response to second-line ARSIs and as a readily testable drug target to improve outcomes for patients with PCa.

## Methods

### Sex as a biological variable.

Our study examined only male patients and animal models, as PCa is a male-specific disease.

### Tumor specimens and sequencing data generation.

WCDT samples were obtained by image-guided fresh-frozen biopsies as previously described (5, 36). DNA and RNA were extracted, and WGS and RNA-Seq libraries were prepared as previously described (5, 36). HMF samples were obtained in a similar fashion; WGS and RNA-Seq data were generated as previously described (88). For each patient, WGS data were also generated from PBMCs to serve as the normal control for somatic DNA analysis.

### WGS data processing and variant calling.

For WCDT samples, sequencing reads were aligned in FASTQ format to the reference human genome GRCh38 with decoy sequences using bwa (89), and somatic variant calling was performed using an updated in-house bioinformatics pipeline as previously reported (5). To analyze SNVs and indels, we used Strelka2 (90) and MuTect2 (91) for variant calling, followed by SnpEff (92) for variant annotation. To analyze CNAs and SVs, we used the GRIDSS/PURPLE/LINX pipeline (62, 93, 94). Variant call sets were compiled, reformatted, and loaded into R for downstream analysis using poppy, our in-house R package. For HMF samples, we converted the original WGS data in CRAM format (95) into BAM files using samtools (96) and then processed them using the same pipeline.

### RNA-Seq data processing and analysis.

Both the WCDT and HMF RNA-Seq data were in FASTQ format. We first quantified gene-level expression using kallisto (97) and then used the abundance calls for downstream analysis. For AR-V7 analysis, we used the transcript-level expression quantified by kallisto (97). We corrected for batch effects using ComBat-Seq (98), and then used DESeq2 (99) for DGE analysis. We performed unpaired analyses to allow the detection of heterogenous gene expression changes and paired analysis (i.e., including patient labels as a covariate in the linear model) to detect the gene expression changes shared among patients. Gene expression values after variance stabilizing transformations (vst in DESeq2) were used for plotting. Tumor purity was estimated by PURPLE (62) whenever WGS data were available or ESTIMATE (100) when only the RNA-Seq data were available and was included as a covariate in the linear model to account for its effect on gene expression analysis. Shrinkage estimators from DESeq2 (99) were used to quantify effect sizes. ssGSEA was performed using the R package GSVA (34).

### Noncoding DNA analysis.

We performed a genome-wide analysis for CNAs over consecutive 1 kb bins. Genomic bins were generated using GenomicRanges (101), and the copy number for each bin was calculated as the weighted average of the intersecting copy number segments reported by PURPLE (62). For genomic regions of interest, we used AME (44) from the MEME suite (102) to search the JASPAR 2022 core vertebrates V2 database (103) for enriched TF binding motifs (using scrambled sequences as controls).

### Public data.

The ECDT dataset (63) was downloaded from cBioPortal (26, 59), including variant calls and fragments per kilobase of transcript per million mapped reads (FPKM) values. Because 2 different RNA-Seq methods (“capture” and “polyA”) were used in ECDT, we followed the example of cBioPortal (26, 59) and reported our reanalysis focusing on *SSTR1* expression and *AR* mutations separately for the 2 methods. RNA-Seq data from the COMBAT-CRPC trial (67) were downloaded from the NCBI’s Gene Expression Omnibus (GEO) database (GEO GSE229555). RNA-Seq data from the SPT preclinical study in enzalutamide-resistant PDX models (68) were downloaded from GEO (GEO GSE124704). cistromeDB (42, 43) and ReMap (45) were queried through their respective web interfaces.

### Cell lines and reagents.

The cell lines used in this study were purchased from the American Type Culture Collection (ATCC). 22Rv1 cells were grown in Gibco RPMI 1640 medium (Thermo Fisher Scientific, A1049101). HEK-293T cells were grown in Gibco DMEM medium (Thermo Fisher Scientific, 11965092). All cell lines were supplemented with 10% FBS (Gibco, Thermo Fisher Scientific, 26140079) and 1× Pen-Strep (Gibco, Thermo Fisher Scientific, 15140-122) and incubated in a 5% CO_2_ humidified chamber at 37°C. Cell line authentications were done at the UC Berkeley DNA Sequencing Facility.

### Plasmids and cell transfection.

To construct expression plasmids, sgRNA targeting *SSTR1* was selected from the CRISPRi V2 library (106) and subcloned into the pLG1 vector (Addgene #217306). 22Rv1 cells with stable overexpression of the dCas9-KRAB construct (a human codon–optimized, nuclease-deficient, catalytically dead Cas9 protein fused to a Krüppel-associated box domain) 22Rv1i was a gift from Luke Gilbert (Arc Institute, Palo Alto, California, USA). The human *SSTR1* (cDNA from Origene, RC207589) overexpression construct was subcloned into pCDH-CMV (Addgene #72265) via Gibson cloning. Cloned plasmids were validated by Sanger sequencing (Primordium Labs and MCLAB South San Francisco, California, USA) and packaged into lentivirus following the Weissman Lab Mega Lentivirus Transfection protocol. 22Rv1 and 22Rv1i at approximately 50% confluence were then transfected with filtered virus and selected with puromycin after 3 days.

### Reverse transcription and quantitative PCR.

Total RNA extractions were performed utilizing Quick-RNA MiniPrep kits (ZymoResearch, R1055) following the manufacturer’s instructions. The quality and quantity of RNA were determined using the NanoDrop OneC Microvolume UV-Vis Spectrophotometer (Thermo Fisher Scientific). cDNA was synthesized using the SuperScript III First-Strand Synthesis System for RT-PCR (Invitrogen, Thermo Fisher Scientific, 18080-051). Quantitative reverse transcription PCR (qRT-PCR) was performed on the QuantStudio 7 Flex System (Life Technologies, Thermo Fisher Scientific). The primers used are listed in Supplemental Table 5 as customized purchases from Integrated DNA Technologies. Expression of target genes was normalized to 18s RNA (internal control) and the control group.

### Western blotting.

SSTR1 Western blotting was performed using the Cell Signaling Technology (CST) antibody (CST 11830) for genetically modified 22Rv1 cells (sgGAL4 and sgSSTR1 for *SSTR1* knockdown; oeGAL4 and oeSSTR1 for *SSTR1* overexpression). Total protein was heated in a 1× dilution of NuPAGE LDS Sample Buffer (Thermo Fisher Scientific, NP0007) at 70°C for 10 minutes. Samples were run in a 4%–12% Bis-Tris SDS-PAGE gel with a Kaleidoscope protein ladder (Bio-Rad, 1610375) for 1 hour and then transferred onto a nitrocellulose membrane. The protein-containing membrane was blocked in an equal mixture of 5% BSA and 5% nonfat milk for 24 hours before incubation overnight in a 1:200 dilution of primary anti-SSTR1 antibody. Samples were washed 3 times in TBS-Tween, incubated for 1 hour in 1:2,000 LI-COR secondary antibody dilution, washed again 3 times in TBS-Tween, and finally imaged on an Odyssey Imager.

### IncuCyte proliferation assay.

22Rv1 cells with stable overexpression, 22Rv1-knockdown cells, and WT 22Rv1 cells were labeled using Nuclight Red lentivirus (Sartorius, 4476) for IncuCyte experiments. Cells were seeded in Falcon 96-well plates (Corning, 353072) at 2,500 cells/well in 100 μL media (3–9 replicates per plate) and imaged for *t*0. Cells were then treated with additional media, 40 μM pasireotide (MedChem Express, HY-16381A), or 10 μM cyclosomatostatin (HY-P1201) and imaged every 24 hours for 7 days. All data points were normalized to T0 and the relevant control group.

### DHT treatment assay.

Cells were seeded at 300,000/well in a 6-well VWR Tissue Culture Plate (Avantor, 10062-892) and allowed to adhere to the surface in a 37°C 5% CO_2_ incubator for 24 hours. DHT was diluted in 1× Dulbecco’s PBS (DPBS) to concentrations of 0.3, 1.0, and 3.0 nM; an equal volume of DMSO corresponding to the maximum volume of DHT added was also diluted in 1× DPBS and utilized as a control. RNA was collected after 48 hours, and qRT-PCR was performed as described above. All samples were normalized to the DMSO control.

### Statistics.

All statistical analyses were performed using R 4.2.0 (104). Fisher’s exact test was used to compare proportions. The Kruskal-Wallis test was used to compare multiple groups. Linear regression was used to analyze quantitative phenotypes. Correlation analyses were performed using Spearman’s method. Survival analysis was performed using the survival package in R (105), and survival data were visualized using the Kaplan-Meier method, with the endpoint being overall survival defined as the time interval from the biopsy analyzed to death from any cause. All statistical tests were 2 sided unless otherwise specified, with a *P* value of less than 0.05 considered statistically significant. Results were corrected for multiple hypothesis testing using Bonferroni’s correction or the Benjamini-Hochberg procedure (FDR) as reported.

### Study approval.

The WCDT samples were obtained through metastatic biopsies as part of a multi-institutional study (ClinicalTrials.gov NCT02432001). The HMF samples were obtained through a study approved by UMC Utrecht (approval no. NL35781.041.11). The studies were conducted in accordance with the Declaration of Helsinki. All individuals provided written informed consent for the collection of tumor biopsies and comprehensive molecular profiling of tumor and germline samples.

### Data availability.

All WCDT data for the paired biopsies are deposited in the public domain. The WGS data accession numbers are as follows: dbGAP:phs001648, EGAS00001006649, EGA00002129194, and EGAS50000000327. The RNA-Seq data accession numbers are as follows: EGAD00001008487, EGAD00001008991, EGAD00001009065, EGA00002236466, and EGA00002166515. The HMF data are available to qualified researchers upon request to the HMF (107). The processed data used to generate all the main and supplemental figures and tables are available in the RData file, which is available upon request. The code used in this manuscript can be obtained upon request from the corresponding author. Supporting data for generating the relevant figures are provided in the Supporting Data Values file.

## Author contributions

XZ, DAQ, MSVDH, and FYF conceived the study. XZ, TF, DV, IY, HL, TL, MS, RS, JK, TS, MZ, AL, TMR, ASW, and AF conducted experiments and formal analysis. NM, RRA, AMB, EJS, NAL, WZ, DAQ, MSVDH, and FYF provided resources. EJS, DAQ, MSVDH, and FYF acquired funding. DAQ, MSVDH, and FYF provided supervision. XZ wrote the manuscript. All authors reviewed and edited the manuscript.

## Supplementary Material

Supplemental data

ICMJE disclosure forms

Unedited blot and gel images

Supplemental table 1

Supplemental table 2

Supporting data values

## Version 1 10/01/2024

Electronic publication

## Version 2 10/03/2024

Affiliations 6-8 corrected. Formatting corrected in abstract.
