# Supplementary material for: Genomic and transcriptomic features of androgen receptor signaling inhibitor resistance in metastatic castration-resistant prostate cancer
Source: J Clin Invest. 2024 Aug 13;134(19):e178604. doi: 10.1172/JCI178604 (PMC11444163; doi:10.1172/JCI178604)
Supplement: Supplemental data [file jci-134-178604-s008.pdf]

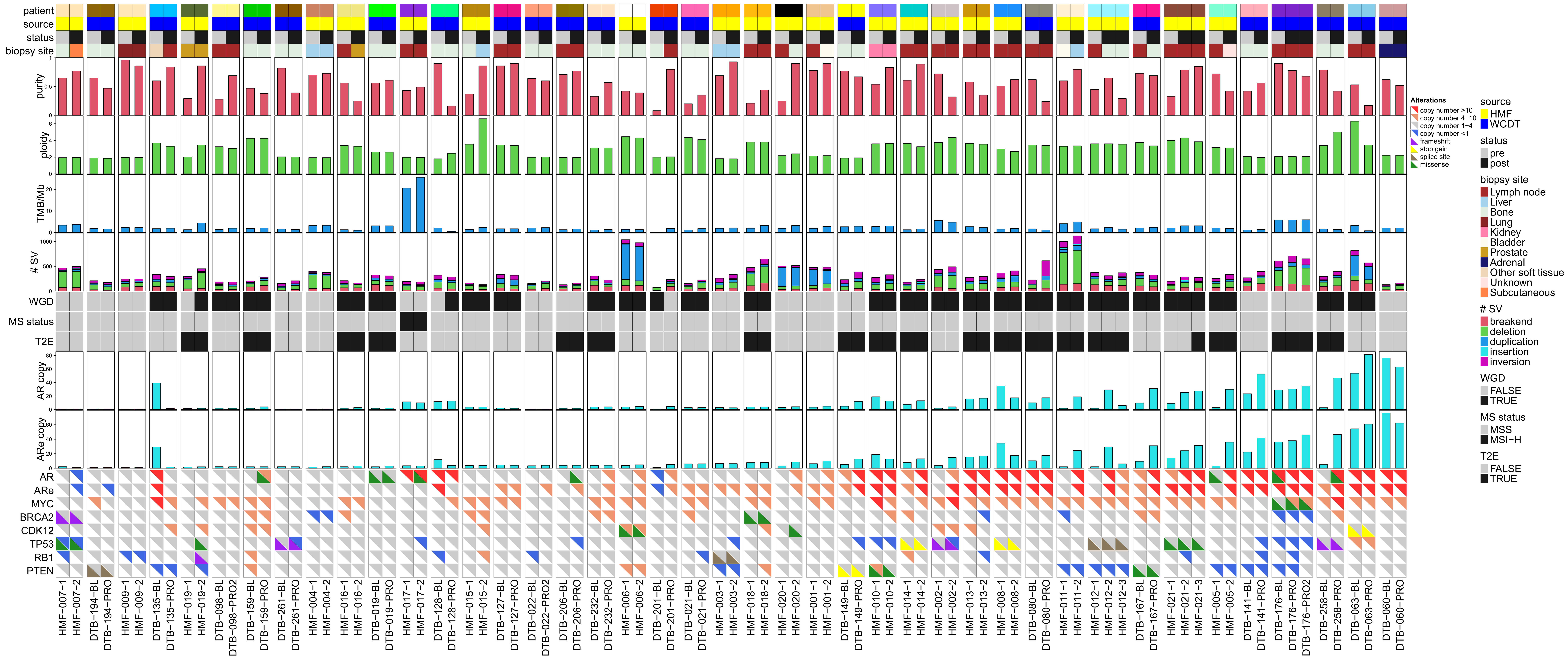

patient
source
status
biopsy site
purity
ploidy
TMB/Mb
SV
WGD
MS status
T2E
AR copy
ARe copy
AR
ARe
MYC
BRCA2
CDK12
TP53
RB1
PTEN
HMF-007-1
HMF-007-2
DTB-194-BL
DTB-194-PRO
HMF-009-1
HMF-009-2
DTB-135-BL
DTB-135-PRO
HMF-019-1
HMF-019-2
DTB-098-BL
DTB-098-PRO2
DTB-159-BL
DTB-159-PRO
DTB-261-BL
DTB-261-PRO
HMF-004-1
HMF-004-2
HMF-016-1
HMF-016-2
DTB-019-BL
DTB-019-PRO
HMF-017-1
HMF-017-2
DTB-128-BL
DTB-128-PRO
HMF-015-1
HMF-015-2
DTB-127-BL
DTB-127-PRO
DTB-022-BL
DTB-022-PRO2
DTB-206-BL
DTB-206-PRO
DTB-232-BL
DTB-232-PRO
HMF-006-1
HMF-006-2
DTB-201-BL
DTB-201-PRO
DTB-021-BL
DTB-021-PRO
HMF-003-1
HMF-003-2
HMF-018-1
HMF-018-2
HMF-020-1
HMF-020-2
HMF-001-1
HMF-001-2
DTB-149-BL
DTB-149-PRO
HMF-010-1
HMF-010-2
HMF-014-1
HMF-014-2
HMF-002-1
HMF-002-2
HMF-013-1
HMF-013-2
HMF-008-1
HMF-008-2
DTB-080-BL
DTB-080-PRO
HMF-011-1
HMF-011-2
HMF-012-1
HMF-012-2
HMF-012-3
DTB-167-BL
DTB-167-PRO
HMF-021-1
HMF-021-2
HMF-021-3
HMF-005-1
HMF-005-2
DTB-141-BL
DTB-141-PRO
DTB-176-BL
DTB-176-PRO
DTB-176-PRO2
DTB-258-BL
DTB-258-PRO
DTB-063-BL
DTB-063-PRO
DTB-060-BL
DTB-060-PRO
Alterations
copy number >10
copy number 4–10
copy number 1–4
copy number <1
frameshift
stop gain
splice site
missense
source
HMF
WCDT
status
pre
post
biopsy site
Lymph node
Liver
Bone
Lung
Kidney
Bladder
Prostate
Adrenal
Other soft tissue
Unknown
Subcutaneous
SV
breakend
deletion
duplication
insertion
inversion
WGD
FALSE
TRUE
MS status
MSS
MSI−H
T2E
FALSE
TRUE

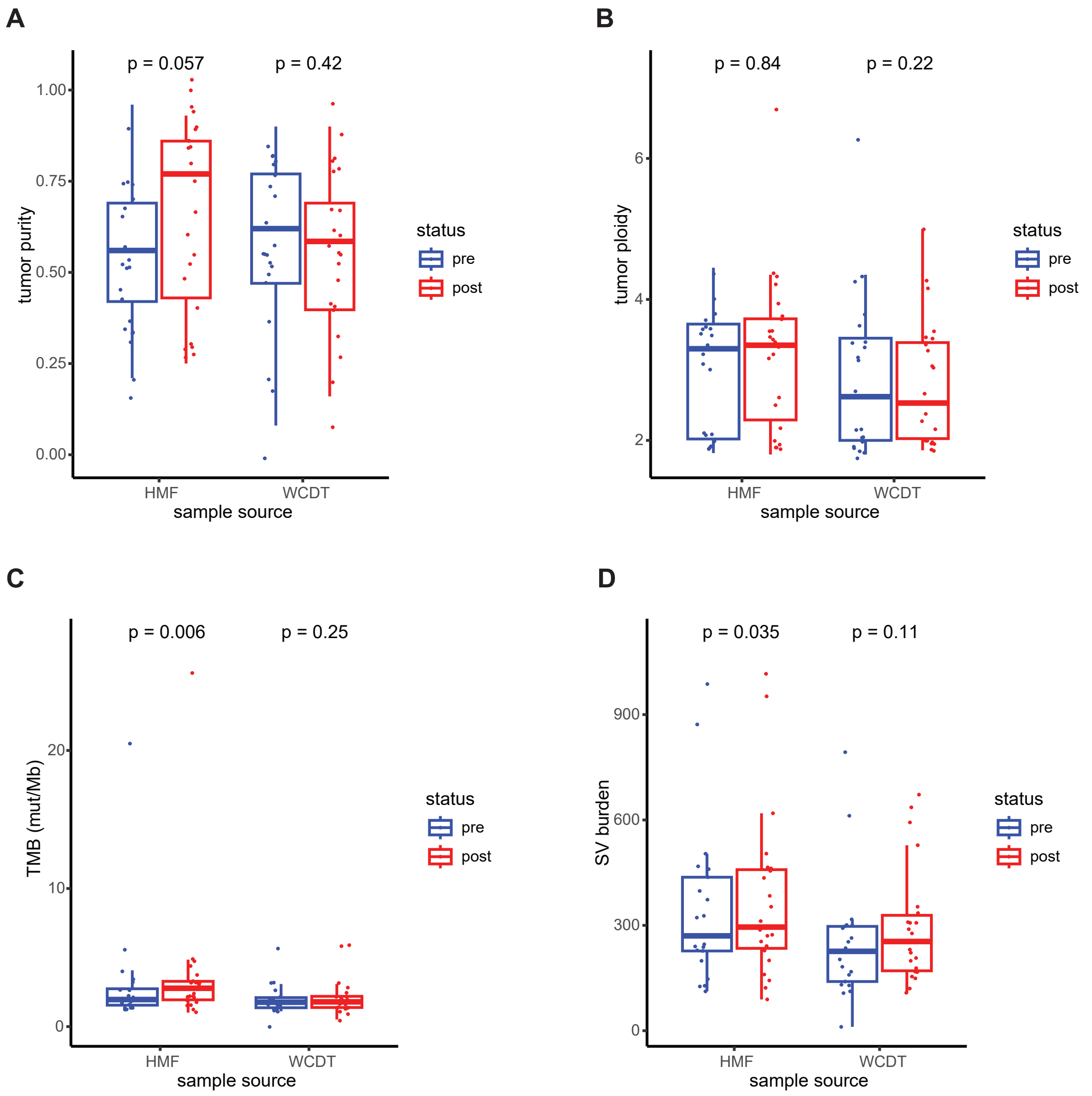
A
p = 0.057
p = 0.42
tumor purity
1.00
0.75
0.50
0.25
0.00
HMF
WCDT
sample source
status
pre
post
B
p = 0.84
p = 0.22
tumor ploidy
6
4
2
HMF
WCDT
sample source
status
pre
post
C
p = 0.006
p = 0.25
TMB (mut/Mb)
20
10
0
HMF
WCDT
sample source
status
pre
post
D
p = 0.035
p = 0.11
SV burden
900
600
300
0
HMF
WCDT
sample source
status
pre
post

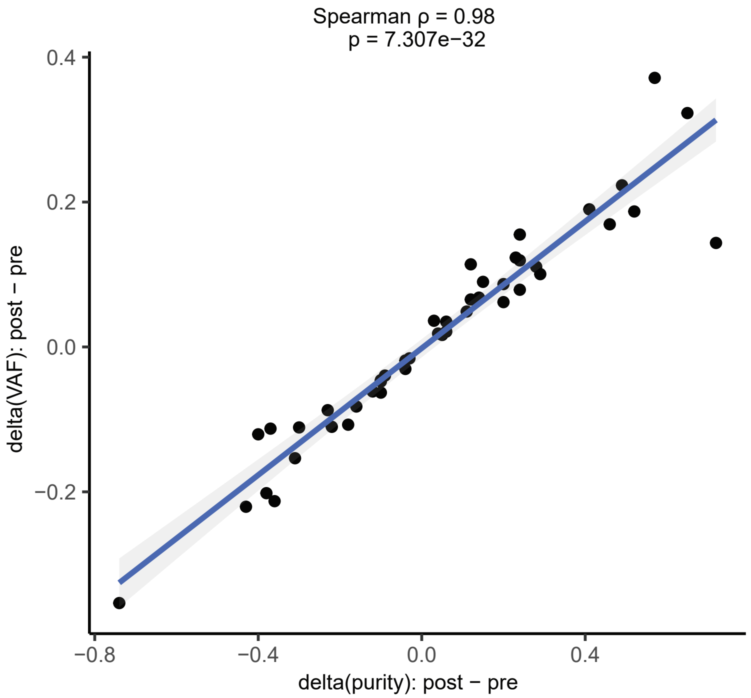
Spearman ρ = 0.98
p = 7.307e−32
0.4
0.2
0.0
−0.2
delta(VAF): post − pre
−0.8
−0.4
0.0
0.4
delta(purity): post − pre

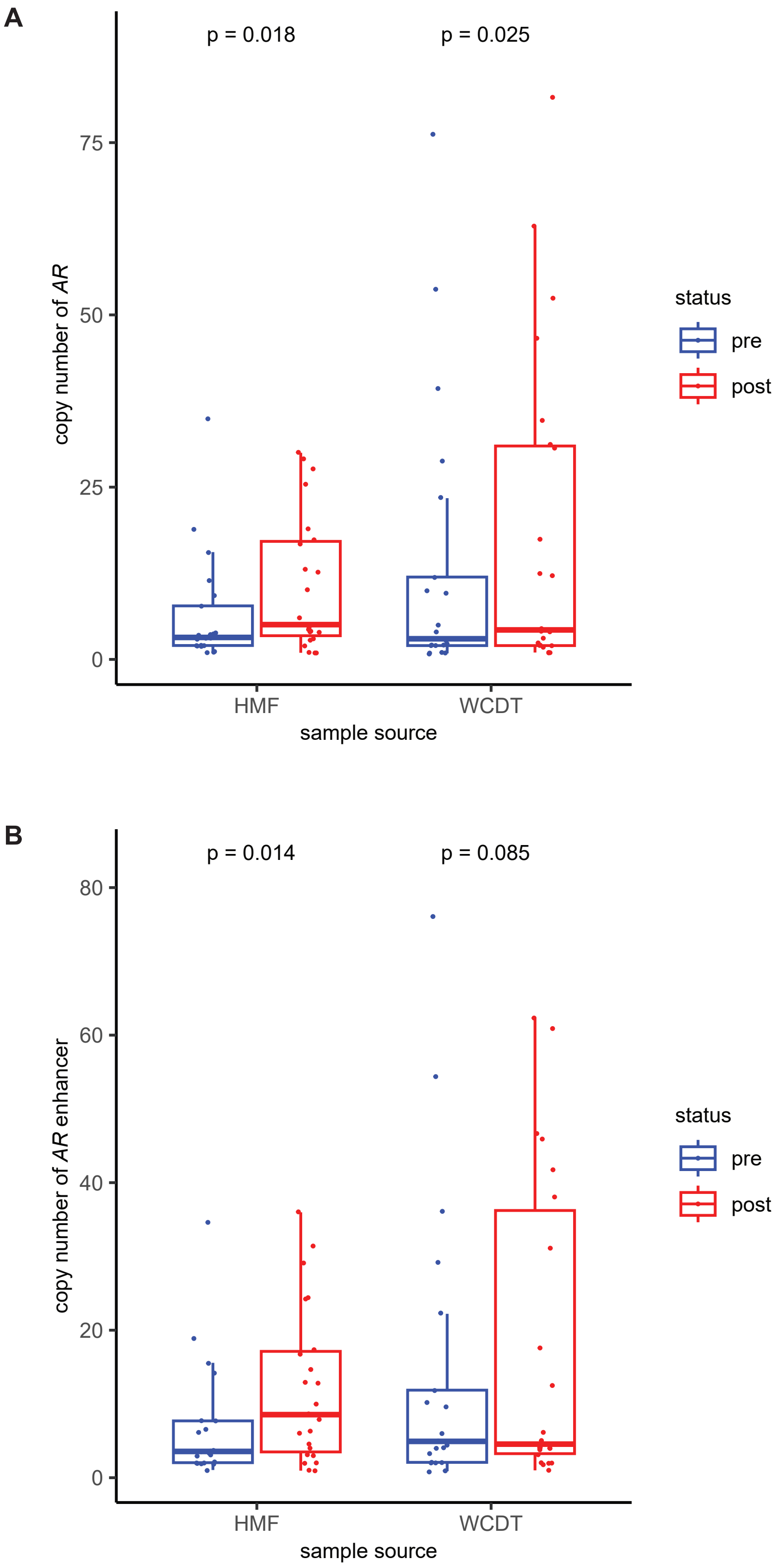
A
p = 0.018
p = 0.025
copy number of AR
0
25
50
75
HMF
WCDT
sample source
status
pre
post
B
p = 0.014
p = 0.085
copy number of AR enhancer
0
20
40
60
80
HMF
WCDT
sample source
status
pre
post

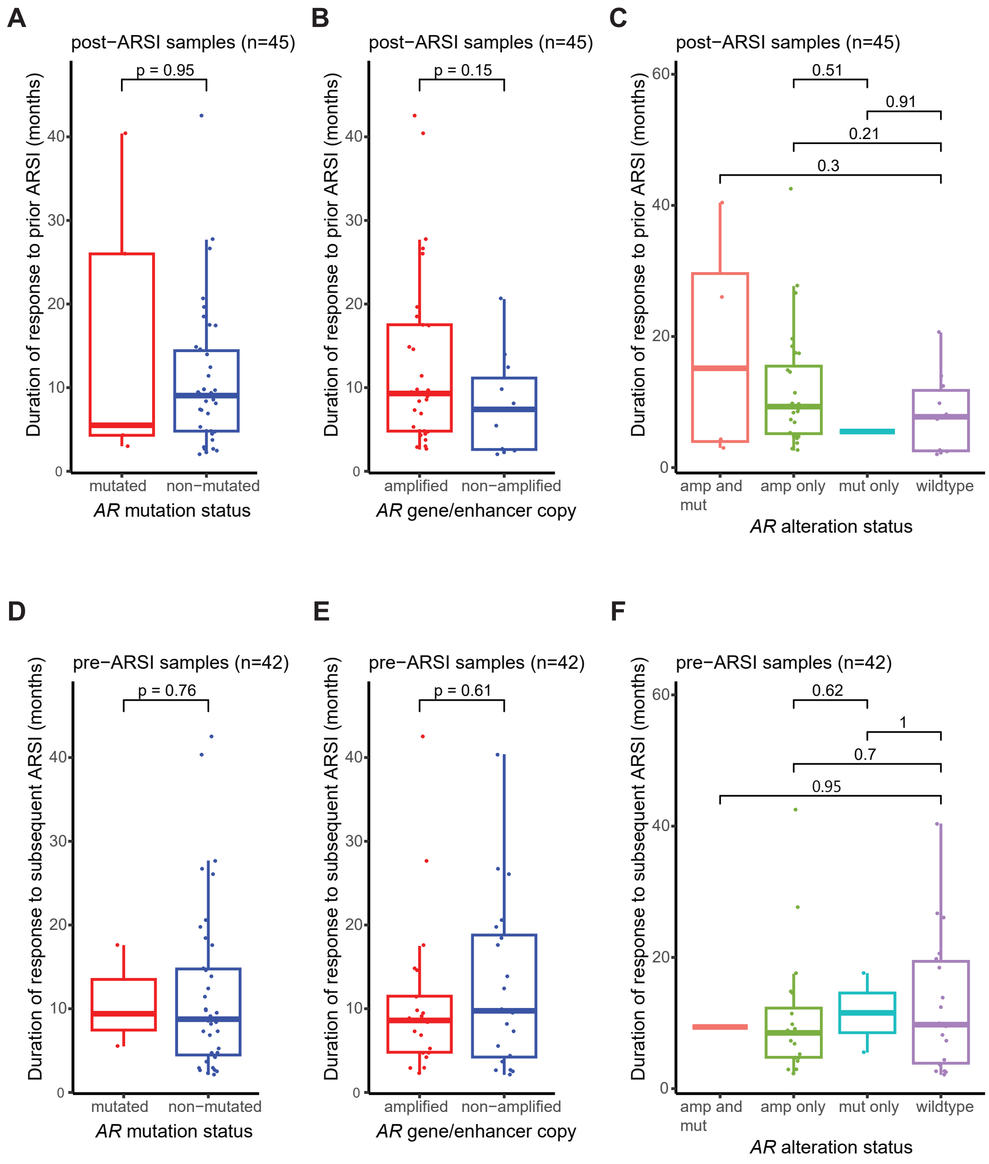
A
post−ARSI samples (n=45)
p = 0.95
Duration of response to prior ARSI (months)
0
10
20
30
40
mutated
non−mutated
AR mutation status
B
post−ARSI samples (n=45)
p = 0.15
Duration of response to prior ARSI (months)
amplified
non−amplified
AR gene/enhancer copy
C
post−ARSI samples (n=45)
0.51
0.91
0.21
0.3
Duration of response to prior ARSI (months)
0
20
40
60
amp and mut
amp only
mut only
wildtype
AR alteration status
D
pre−ARSI samples (n=42)
p = 0.76
Duration of response to subsequent ARSI (months)
mutated
non−mutated
AR mutation status
E
pre−ARSI samples (n=42)
p = 0.61
Duration of response to subsequent ARSI (months)
amplified
non−amplified
AR gene/enhancer copy
F
pre−ARSI samples (n=42)
0.62
1
0.7
0.95
Duration of response to subsequent ARSI (months)
amp and mut
amp only
mut only
wildtype
AR alteration status

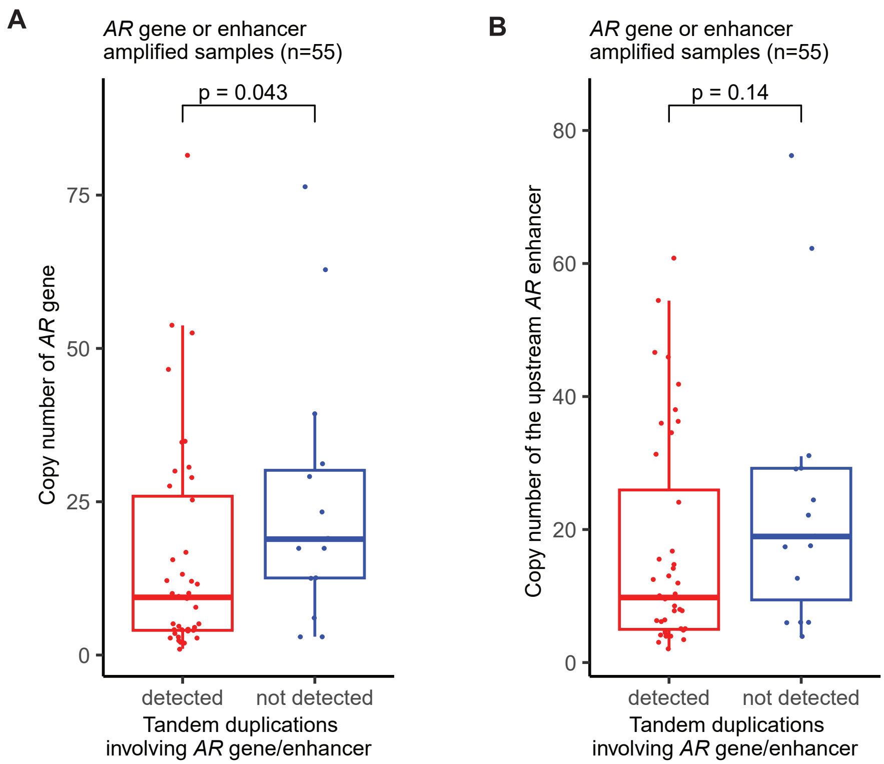
A
AR gene or enhancer
amplified samples (n=55)
p = 0.043
Copy number of AR gene
0
25
50
75
detected
not detected
Tandem duplications
involving AR gene/enhancer
B
AR gene or enhancer
amplified samples (n=55)
p = 0.14
Copy number of the upstream AR enhancer
0
20
40
60
80
detected
not detected
Tandem duplications
involving AR gene/enhancer

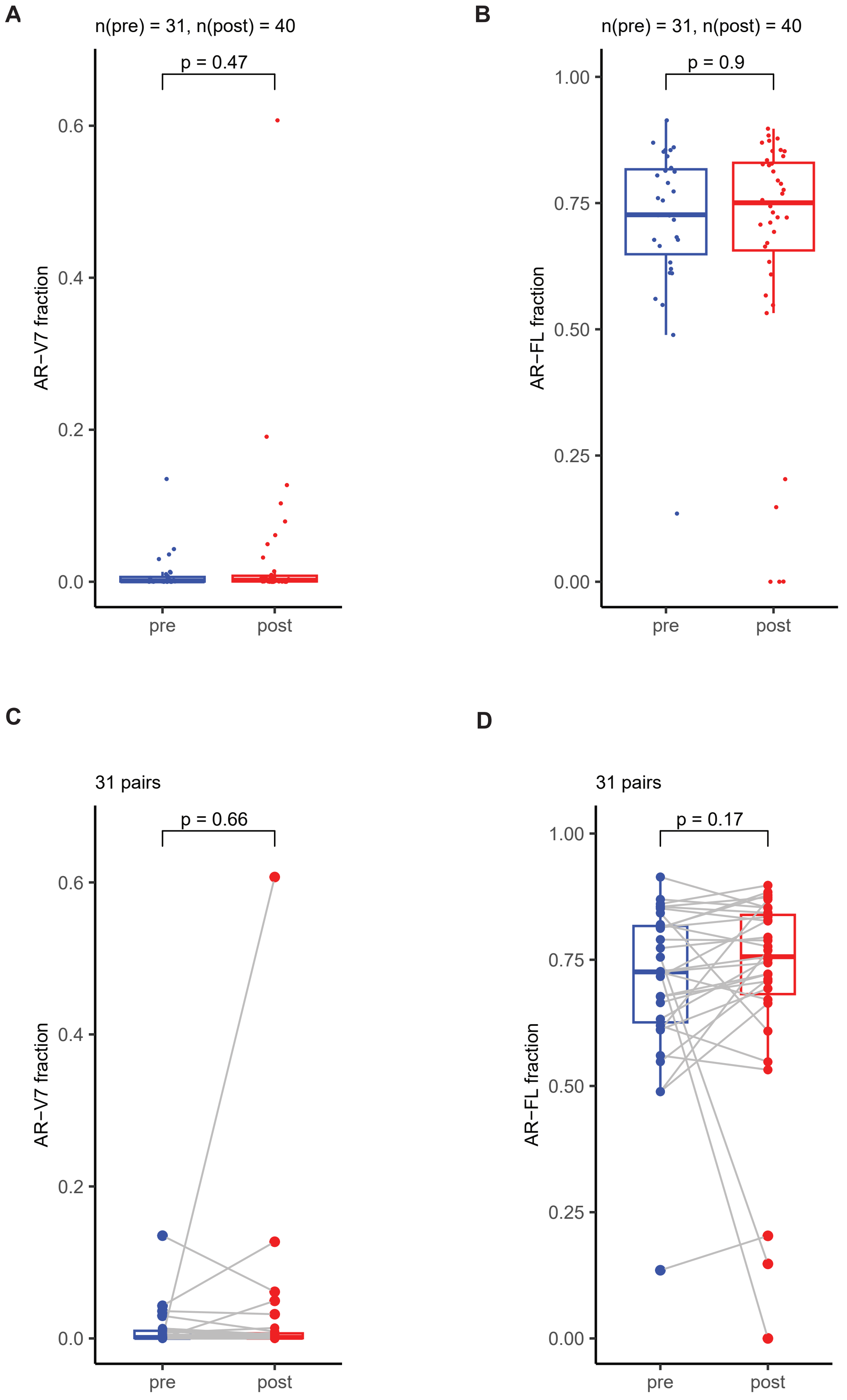
A
n(pre) = 31, n(post) = 40
p = 0.47
AR−V7 fraction
0.0
0.2
0.4
0.6
pre
post
B
n(pre) = 31, n(post) = 40
p = 0.9
AR−FL fraction
0.00
0.25
0.50
0.75
1.00
pre
post
C
31 pairs
p = 0.66
AR−V7 fraction
0.0
0.2
0.4
0.6
pre
post
D
31 pairs
p = 0.17
AR−FL fraction
0.00
0.25
0.50
0.75
1.00
pre
post

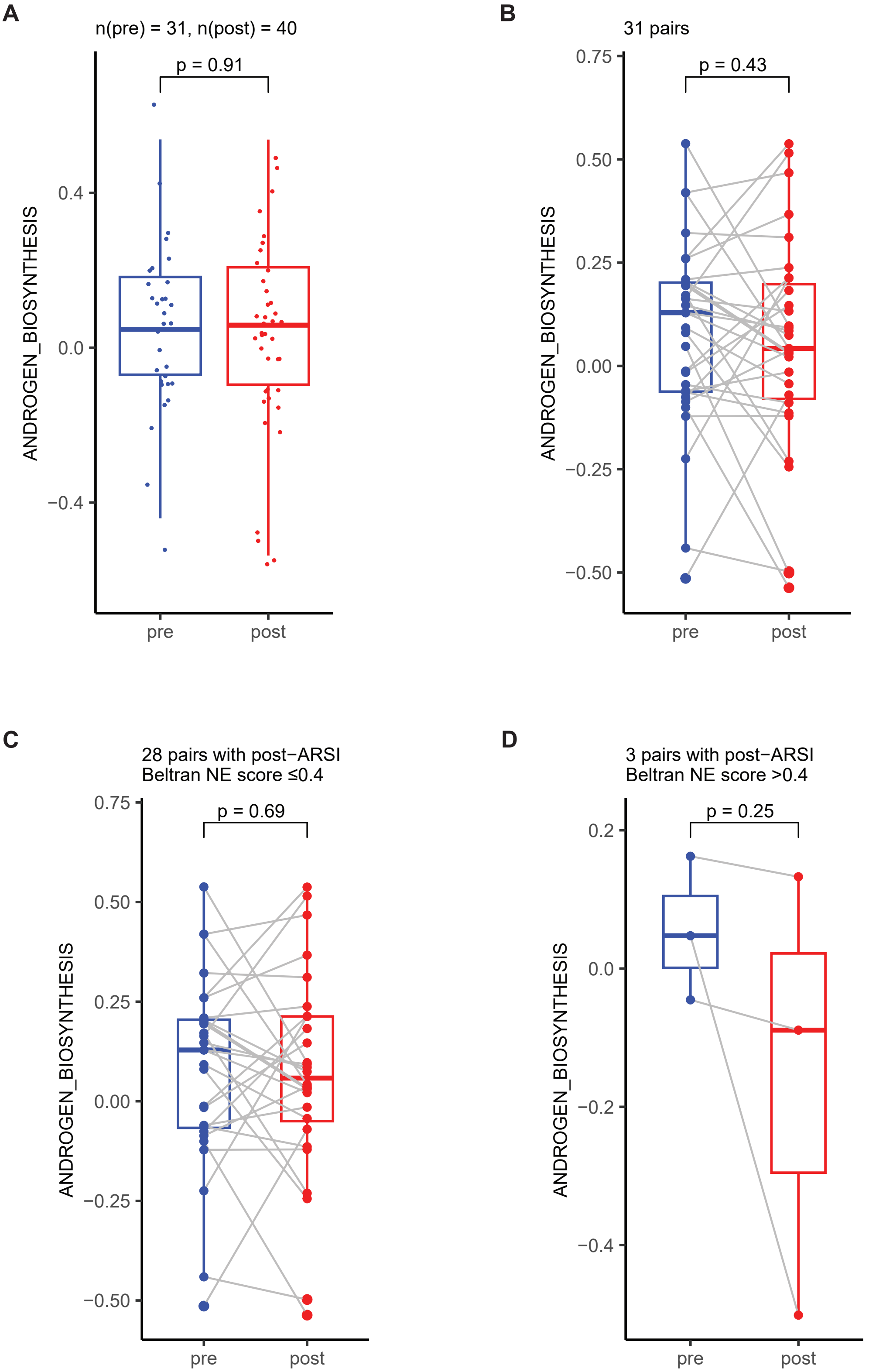

A
n(pre) = 31, n(post) = 40
p = 0.91
ANDROGEN_BIOSYNTHESIS
0.4
0.0
−0.4
pre
post
B
31 pairs
p = 0.43
ANDROGEN_BIOSYNTHESIS
0.75
0.50
0.25
0.00
−0.25
−0.50
pre
post
C
28 pairs with post−ARSI
Beltran NE score ≤0.4
p = 0.69
ANDROGEN_BIOSYNTHESIS
0.75
0.50
0.25
0.00
−0.25
−0.50
pre
post
D
3 pairs with post−ARSI
Beltran NE score >0.4
p = 0.25
ANDROGEN_BIOSYNTHESIS
0.2
0.0
−0.2
−0.4
pre
post

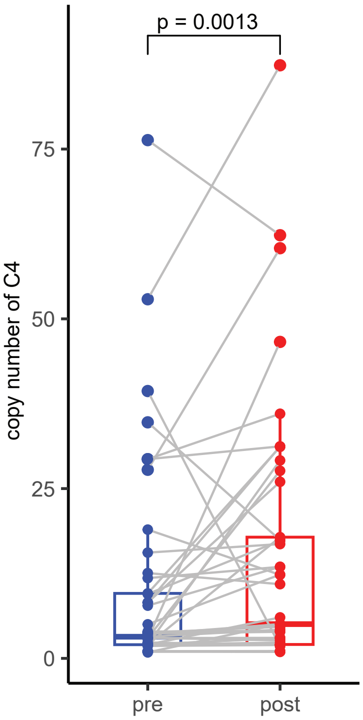

p = 0.0013
copy number of C4
75
50
25
0
pre
post

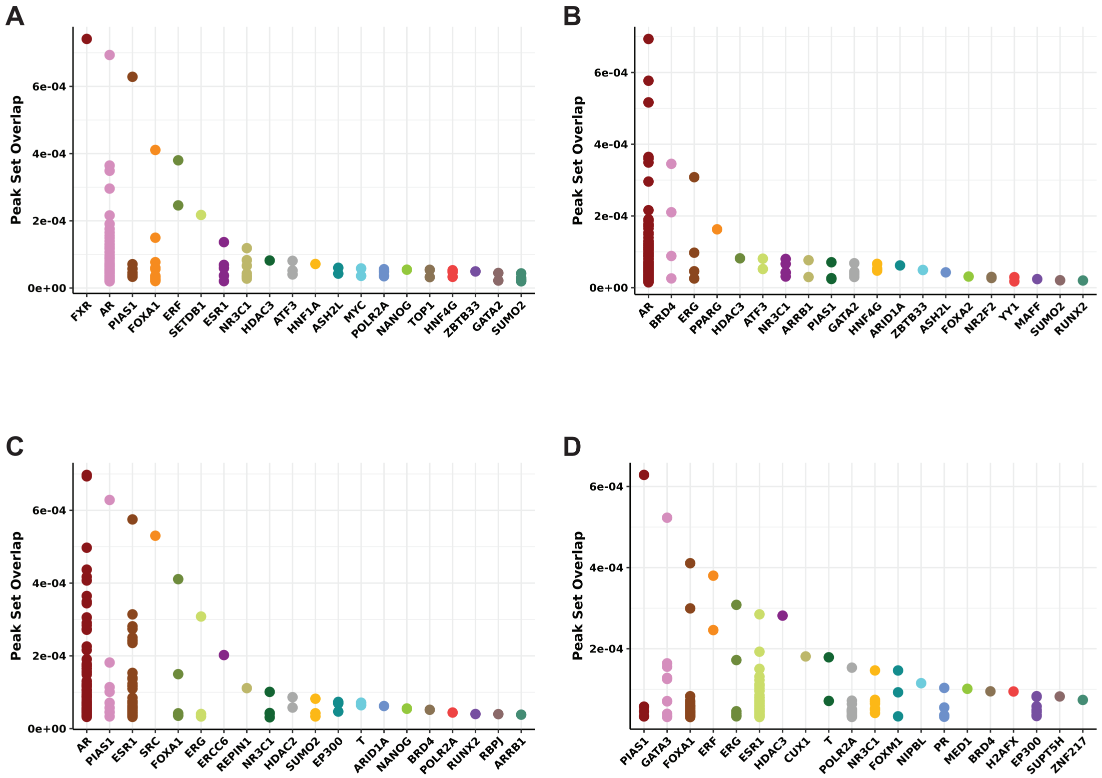

A
B
C
D
Peak Set Overlap
0e+00
2e-04
4e-04
6e-04
FXR AR PIAS1 FOXA1 ERF SETDB1 ESR1 NR3C1 HDAC3 ATF3 HNF1A ASH2L MYC POLR2A NANOG TOP1 HNF4G ZBTB33 GATA2 SUMO2
AR BRD4 ERG PPARG HDAC3 ATF3 NR3C1 ARRB1 PIAS1 GATA2 HNF4G ARID1A ZBTB33 ASH2L FOXA2 NR2F2 YY1 MAFF SUMO2 RUNX2
AR PIAS1 ESR1 SRC FOXA1 ERG ERCC6 REPIN1 NR3C1 HDAC2 SUMO2 EP300 T ARID1A NANOG BRD4 POLR2A RUNX2 RBPJ ARRB1
PIAS1 GATA3 FOXA1 ERF ERG ESR1 HDAC3 CUX1 T POLR2A NR3C1 FOXM1 NIPBL PR MED1 BRD4 H2AFX EP300 SUPT5H ZNF217

A

Logo	Database	ID	Alt ID	*p*-value	*E*-value	TP Thresh	TP (%)	FP (%)
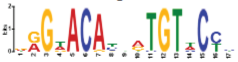	JASPAR2022 CORE vertebrates redundant v2	MA0113.3	NR3C1	6.06e-4	7.30e-1	66.22	2 (66.7%)	2 (0.2%)
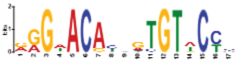	JASPAR2022 CORE vertebrates redundant v2	MA0007.3	Ar	6.77e-4	8.16e-1	66.82	2 (66.7%)	2 (0.2%)
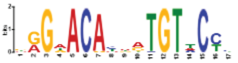	JASPAR2022 CORE vertebrates redundant v2	MA0727.1	NR3C2	1.19e-3	1.43e0	51.11	2 (66.7%)	3 (0.3%)
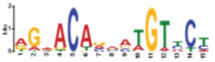	JASPAR2022 CORE vertebrates redundant v2	MA0113.2	NR3C1	7.60e-3	9.15e0	134.31	2 (66.7%)	2 (0.2%)

B

Logo	Database	ID	Alt ID	*p*-value	*E*-value	TP Thresh	TP (%)	FP (%)
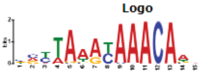	JASPAR2022 CORE vertebrates redundant v2	MA1487.1	FOXE1	2.77e-4	3.34e-1	1.45	4 (100.0%)	45 (4.5%)
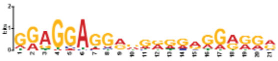	JASPAR2022 CORE vertebrates redundant v2	MA0528.1	ZNF263	9.72e-4	1.17e0	4.56	4 (100.0%)	49 (4.9%)
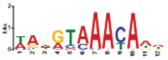	JASPAR2022 CORE vertebrates redundant v2	MA0846.1	FOXC2	1.43e-3	1.73e0	3.45	4 (100.0%)	50 (5.0%)
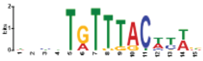	JASPAR2022 CORE vertebrates redundant v2	MA0148.3	FOXA1	1.59e-3	1.91e0	4.49	4 (100.0%)	53 (5.3%)
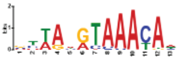	JASPAR2022 CORE vertebrates redundant v2	MA0847.2	FOXD2	1.94e-3	2.33e0	1.99	4 (100.0%)	62 (6.2%)
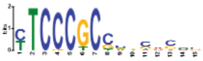	JASPAR2022 CORE vertebrates redundant v2	MA0469.1	E2F3	5.66e-3	6.82e0	8.16	2 (50.0%)	5 (0.5%)

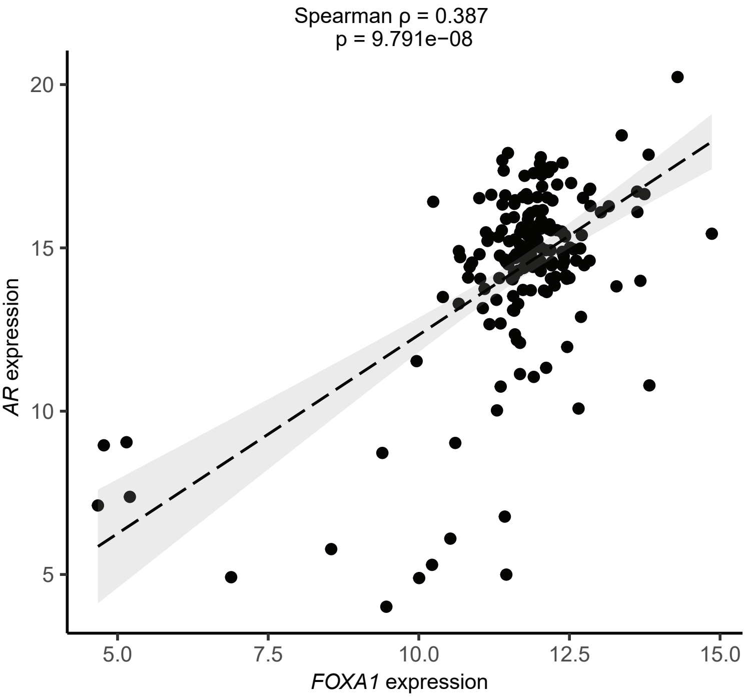

Spearman ρ = 0.387
p = 9.791e−08
20
15
10
5
AR expression
5.0
7.5
10.0
12.5
15.0
FOXA1 expression

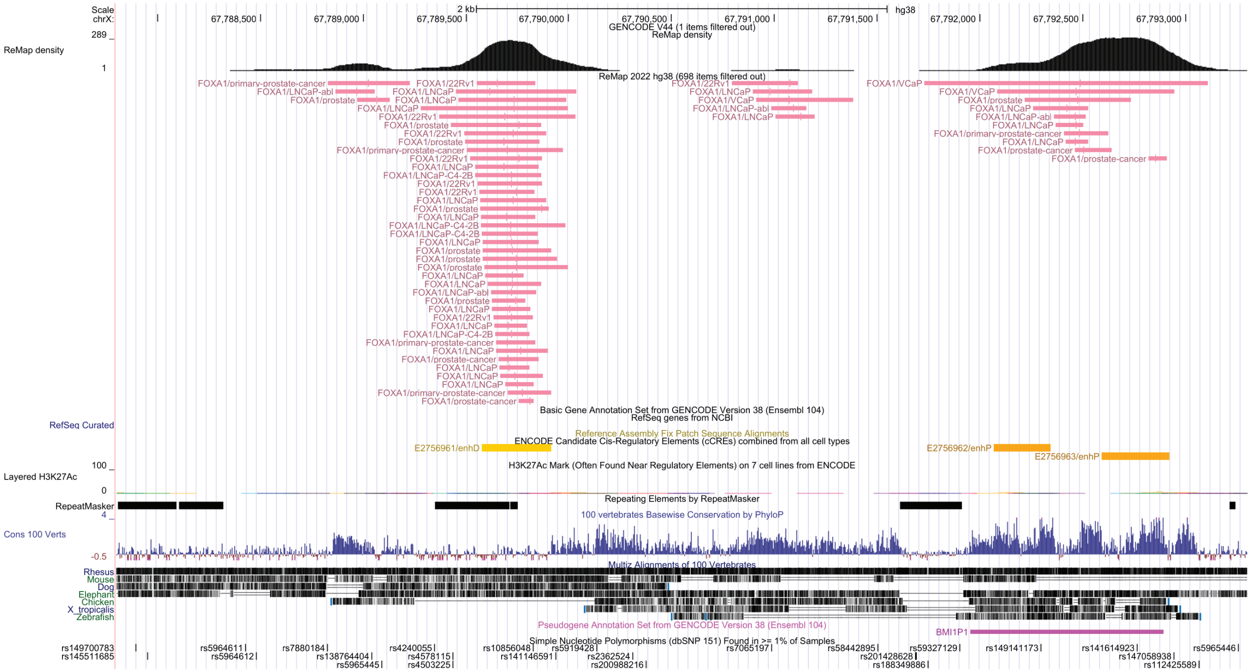
Scale
chrX:
2 kb
hg38
67,788,500
67,789,000
67,789,500
67,790,000
67,790,500
67,791,000
67,791,500
67,792,000
67,792,500
67,793,000
GENCODE V44 (1 items filtered out)
ReMap density
289 _
ReMap density
1
ReMap 2022 hg38 (698 items filtered out)
FOXA1/primary-prostate-cancer
FOXA1/LNCaP-abl
FOXA1/prostate
FOXA1/22Rv1
FOXA1/LNCaP
FOXA1/LNCaP-C4-2B
FOXA1/VCaP
FOXA1/prostate-cancer
Basic Gene Annotation Set from GENCODE Version 38 (Ensembl 104)
RefSeq genes from NCBI
RefSeq Curated
Reference Assembly Fix Patch Sequence Alignments
ENCODE Candidate Cis-Regulatory Elements (cCREs) combined from all cell types
E2756961/enhD
E2756962/enhP
E2756963/enhP
100 _
H3K27Ac Mark (Often Found Near Regulatory Elements) on 7 cell lines from ENCODE
Layered H3K27Ac
0
Repeating Elements by RepeatMasker
RepeatMasker
4 _
100 vertebrates Basewise Conservation by PhyloP
Cons 100 Verts
-0.5
Multiz Alignments of 100 Vertebrates
Rhesus
Mouse
Dog
Elephant
Chicken
X_tropicalis
Zebrafish
Pseudogene Annotation Set from GENCODE Version 38 (Ensembl 104)
BMI1P1
Simple Nucleotide Polymorphisms (dbSNP 151) Found in >= 1% of Samples
rs149700783
rs145511685
rs5964611
rs5964612
rs7880184
rs138764404
rs5965445
rs4240055
rs4578115
rs4503225
rs10856048
rs141146591
rs5919428
rs2362524
rs200988216
rs7065197
rs58442895
rs201428628
rs188349886
rs59327129
rs149141173
rs141614923
rs147058938
rs112425589
rs5965446

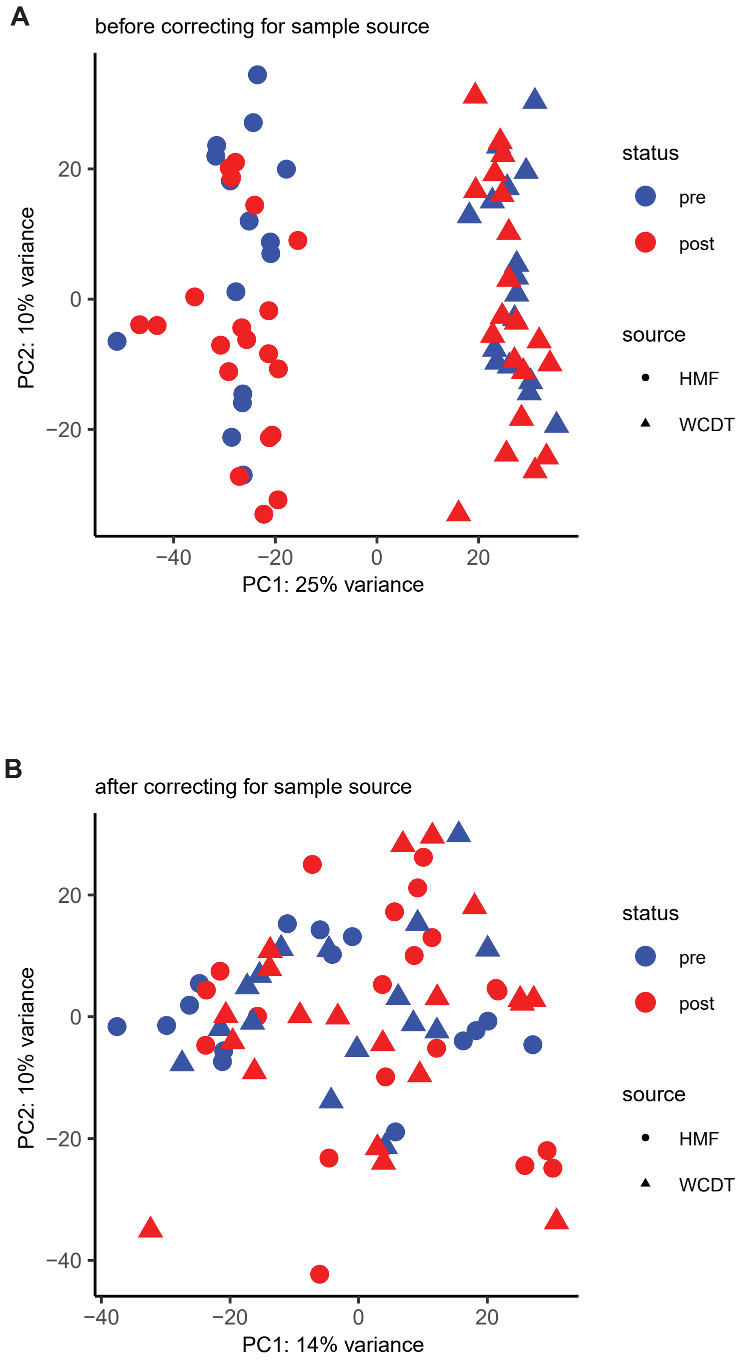
A
before correcting for sample source
PC2: 10% variance
PC1: 25% variance
20
0
−20
−40
−20
0
20
status
pre
post
source
HMF
WCDT
B
after correcting for sample source
PC2: 10% variance
PC1: 14% variance
20
0
−20
−40
−40
−20
0
20
status
pre
post
source
HMF
WCDT

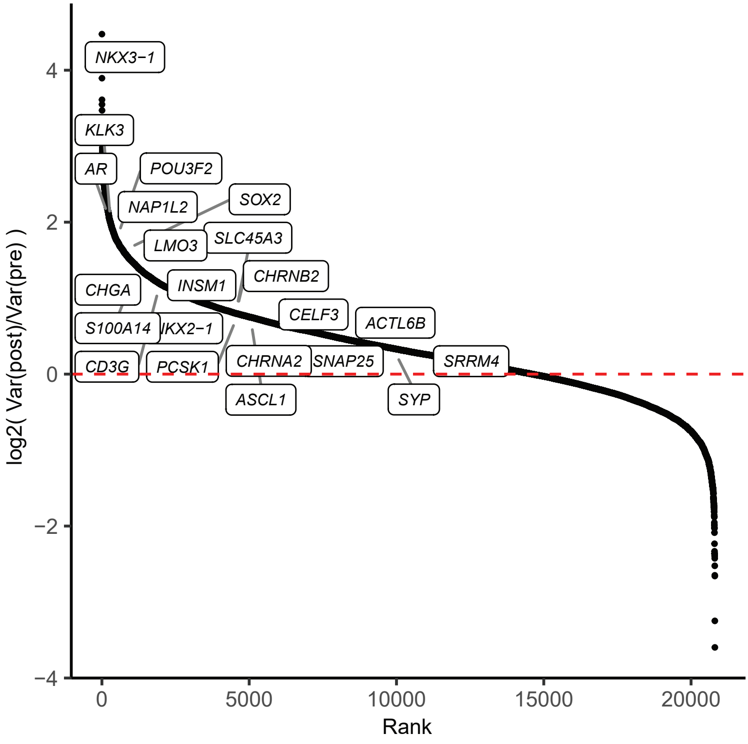

NKX3−1
KLK3
AR
POU3F2
NAP1L2
SOX2
LMO3
SLC45A3
CHRNB2
CHGA
INSM1
CELF3
ACTL6B
S100A14
NKX2−1
CD3G
PCSK1
CHRNA2
SNAP25
SRRM4
ASCL1
SYP
4
2
0
−2
−4
log2(Var(post)/Var(pre))
0
5000
10000
15000
20000
Rank

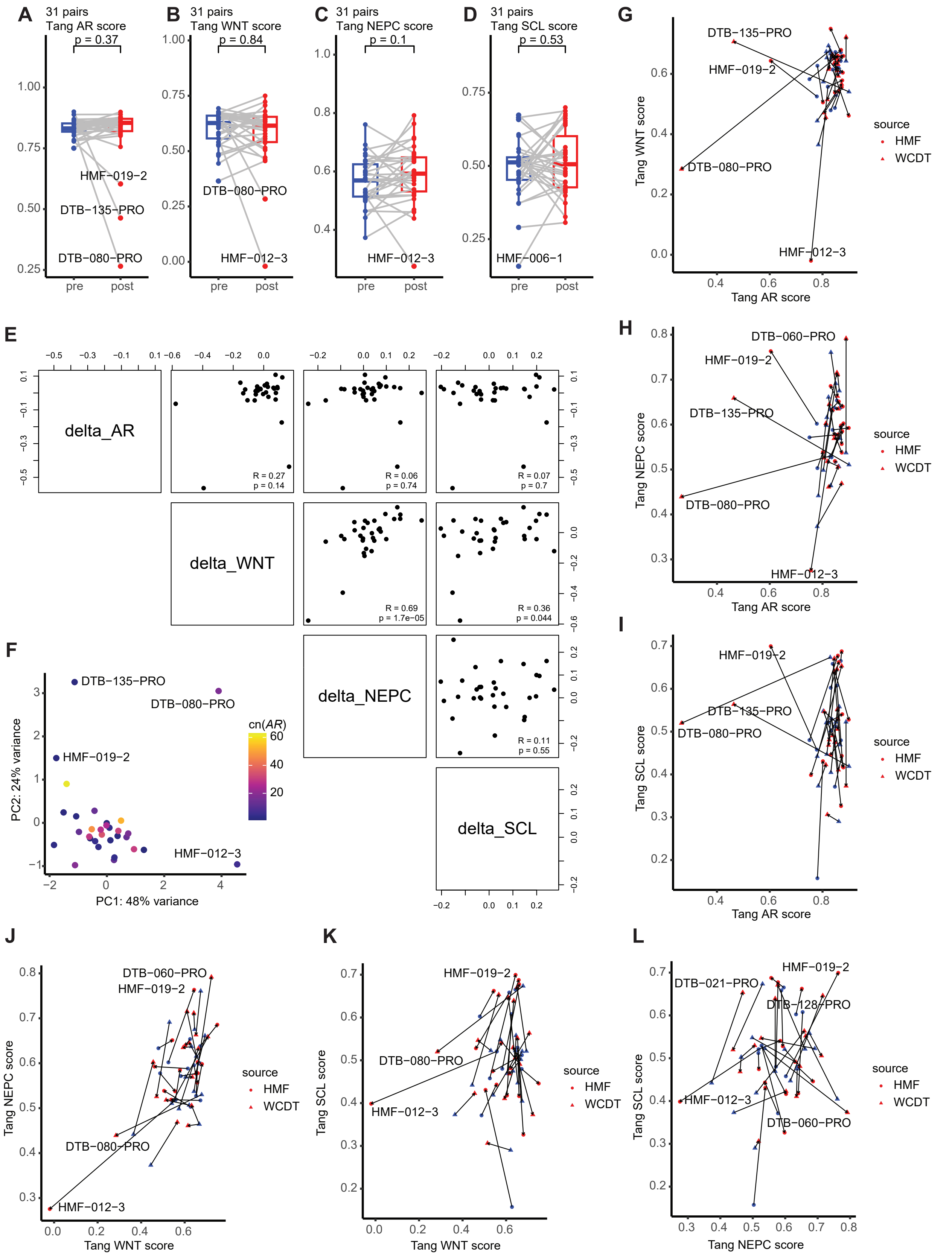
A
31 pairs
Tang AR score
p = 0.37
HMF−019−2
DTB−135−PRO
DTB−080−PRO
pre
post
B
31 pairs
Tang WNT score
p = 0.84
DTB−080−PRO
HMF−012−3
C
31 pairs
Tang NEPC score
p = 0.1
HMF−012−3
D
31 pairs
Tang SCL score
p = 0.53
HMF−006−1
G
DTB−135−PRO
HMF−019−2
DTB−080−PRO
HMF−012−3
Tang WNT score
Tang AR score
source
HMF
WCDT
E
delta_AR
delta_WNT
delta_NEPC
delta_SCL
R = 0.27
p = 0.14
R = 0.06
p = 0.74
R = 0.07
p = 0.7
R = 0.69
p = 1.7e−05
R = 0.36
p = 0.044
R = 0.11
p = 0.55
H
DTB−060−PRO
HMF−019−2
DTB−135−PRO
DTB−080−PRO
HMF−012−3
Tang NEPC score
Tang AR score
I
HMF−019−2
DTB−135−PRO
DTB−080−PRO
Tang SCL score
Tang AR score
F
DTB−135−PRO
DTB−080−PRO
HMF−019−2
HMF−012−3
cn(AR)
PC2: 24% variance
PC1: 48% variance
J
DTB−060−PRO
HMF−019−2
DTB−080−PRO
HMF−012−3
Tang NEPC score
Tang WNT score
K
HMF−019−2
DTB−080−PRO
HMF−012−3
Tang SCL score
Tang WNT score
L
HMF−019−2
DTB−021−PRO
DTB−128−PRO
HMF−012−3
DTB−060−PRO
Tang SCL score
Tang NEPC score

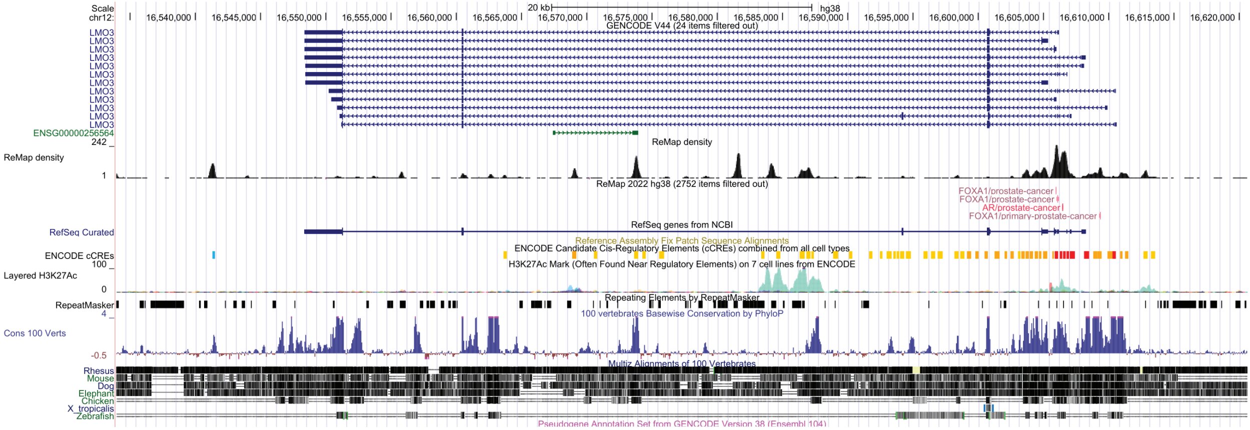
Scale
chr12:
20 kb
hg38
16,540,000
16,545,000
16,550,000
16,555,000
16,560,000
16,565,000
16,570,000
16,575,000
16,580,000
16,585,000
16,590,000
16,595,000
16,600,000
16,605,000
16,610,000
16,615,000
16,620,000
GENCODE V44 (24 items filtered out)
LMO3
ENSG00000256564
ReMap density
242
1
ReMap 2022 hg38 (2752 items filtered out)
FOXA1/prostate-cancer
FOXA1/prostate-cancer
AR/prostate-cancer
FOXA1/primary-prostate-cancer
RefSeq genes from NCBI
RefSeq Curated
Reference Assembly Fix Patch Sequence Alignments
ENCODE Candidate Cis-Regulatory Elements (cCREs) combined from all cell types
ENCODE cCREs
100
H3K27Ac Mark (Often Found Near Regulatory Elements) on 7 cell lines from ENCODE
Layered H3K27Ac
0
Repeating Elements by RepeatMasker
RepeatMasker
4
100 vertebrates Basewise Conservation by PhyloP
Cons 100 Verts
-0.5
Multiz Alignments of 100 Vertebrates
Rhesus
Mouse
Dog
Elephant
Chicken
X_tropicalis
Zebrafish
Pseudogene Annotation Set from GENCODE Version 38 (Ensembl 104)

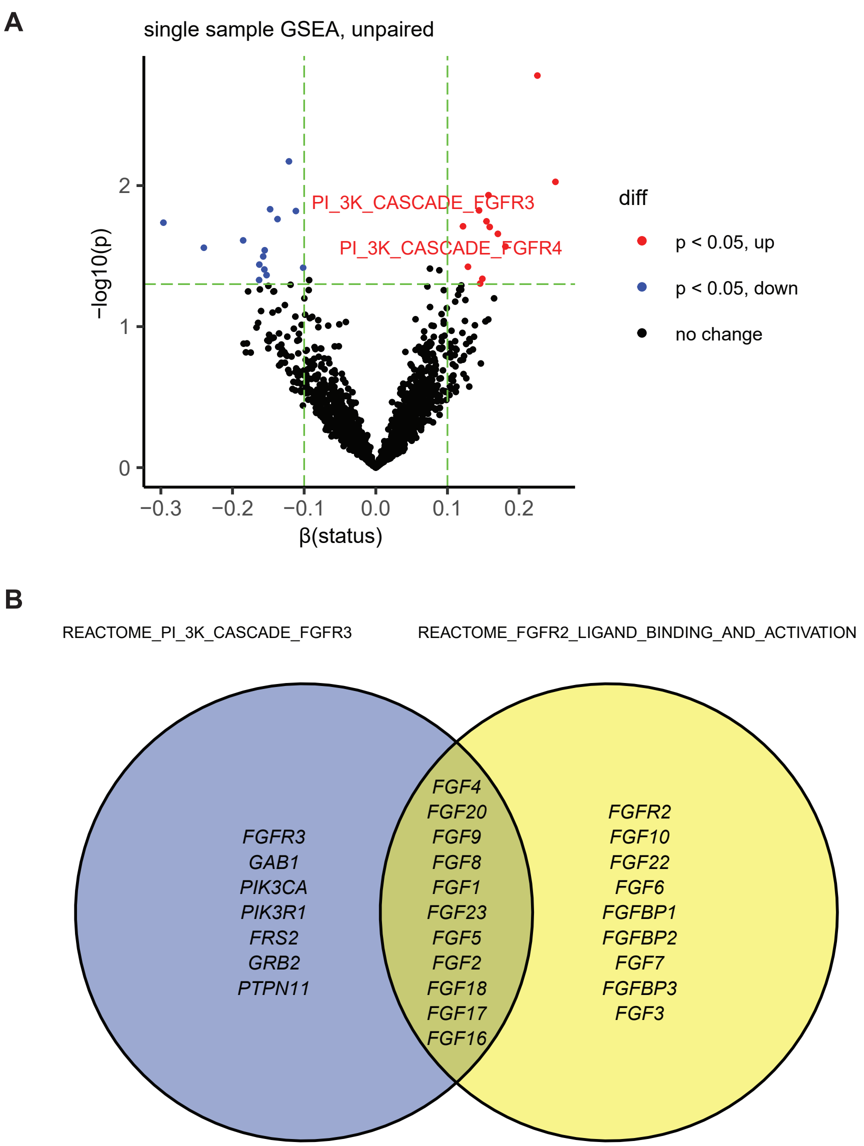
A
single sample GSEA, unpaired
PI_3K_CASCADE_FGFR3
PI_3K_CASCADE_FGFR4
−log10(p)
0
1
2
−0.3
−0.2
−0.1
0.0
0.1
0.2
β(status)
diff
p < 0.05, up
p < 0.05, down
no change
B
REACTOME_PI_3K_CASCADE_FGFR3
REACTOME_FGFR2_LIGAND_BINDING_AND_ACTIVATION
FGFR3
GAB1
PIK3CA
PIK3R1
FRS2
GRB2
PTPN11
FGF4
FGF20
FGF9
FGF8
FGF1
FGF23
FGF5
FGF2
FGF18
FGF17
FGF16
FGFR2
FGF10
FGF22
FGF6
FGFBP1
FGFBP2
FGF7
FGFBP3
FGF3

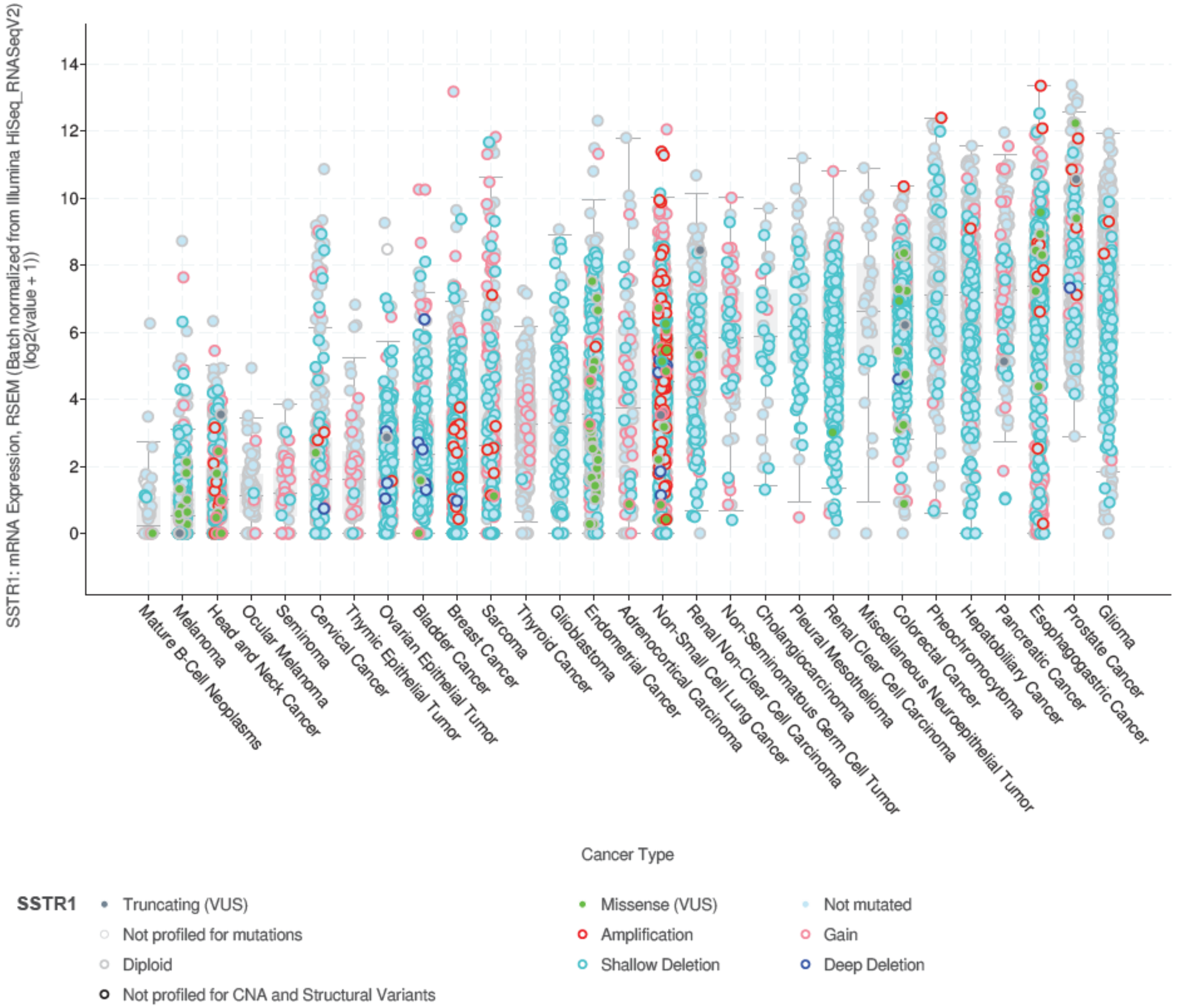

SSTR1: mRNA Expression, RSEM (Batch normalized from Illumina HiSeq_RNASeqV2) (log2(value + 1))
14
12
10
8
6
4
2
0
Mature B-Cell Neoplasms
Melanoma
Head and Neck Cancer
Ocular Melanoma
Seminoma
Cervical Cancer
Thymic Epithelial Tumor
Ovarian Epithelial Tumor
Bladder Cancer
Breast Cancer
Sarcoma
Thyroid Cancer
Glioblastoma
Endometrial Cancer
Adrenocortical Carcinoma
Non-Small Cell Lung Cancer
Renal Non-Clear Cell Carcinoma
Non-Seminomatous Germ Cell Tumor
Cholangiocarcinoma
Pleural Mesothelioma
Renal Clear Cell Carcinoma
Miscellaneous Neuroepithelial Tumor
Colorectal Cancer
Pheochromocytoma
Hepatobiliary Cancer
Pancreatic Cancer
Esophagogastric Cancer
Prostate Cancer
Glioma
Cancer Type
SSTR1
Truncating (VUS)
Not profiled for mutations
Diploid
Not profiled for CNA and Structural Variants
Missense (VUS)
Amplification
Shallow Deletion
Not mutated
Gain
Deep Deletion

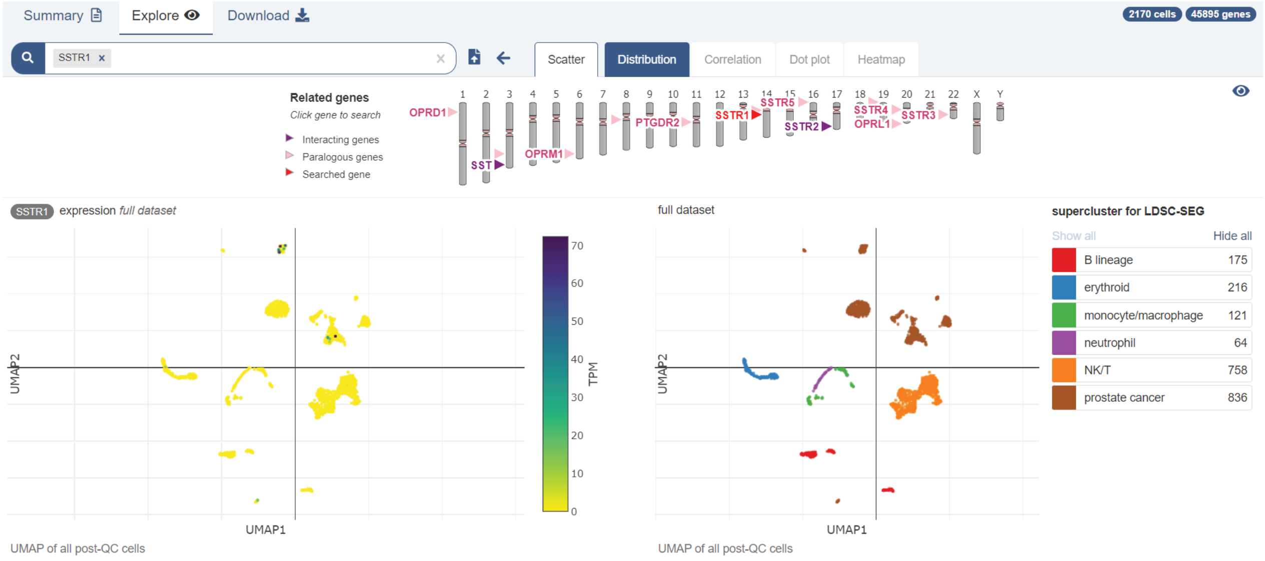
Summary
Explore
Download
2170 cells
45895 genes
SSTR1
Scatter
Distribution
Correlation
Dot plot
Heatmap
Related genes
Click gene to search
Interacting genes
Paralogous genes
Searched gene
OPRD1
SST
OPRM1
PTGDR2
SSTR1
SSTR5
SSTR2
SSTR4
OPRL1
SSTR3
SSTR1 expression full dataset
UMAP2
UMAP1
TPM
UMAP of all post-QC cells
full dataset
UMAP2
UMAP1
UMAP of all post-QC cells
supercluster for LDSC-SEG
Show all
Hide all
B lineage 175
erythroid 216
monocyte/macrophage 121
neutrophil 64
NK/T 758
prostate cancer 836

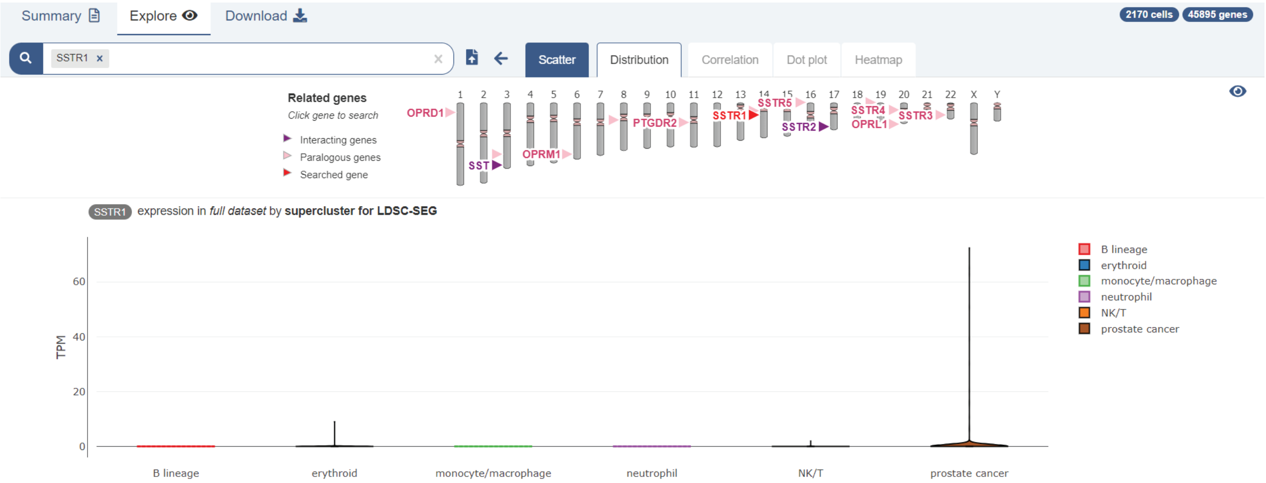
Summary
Explore
Download
2170 cells
45895 genes
SSTR1
Scatter
Distribution
Correlation
Dot plot
Heatmap
Related genes
Click gene to search
Interacting genes
Paralogous genes
Searched gene
SSTR1 expression in full dataset by supercluster for LDSC-SEG
TPM
B lineage
erythroid
monocyte/macrophage
neutrophil
NK/T
prostate cancer

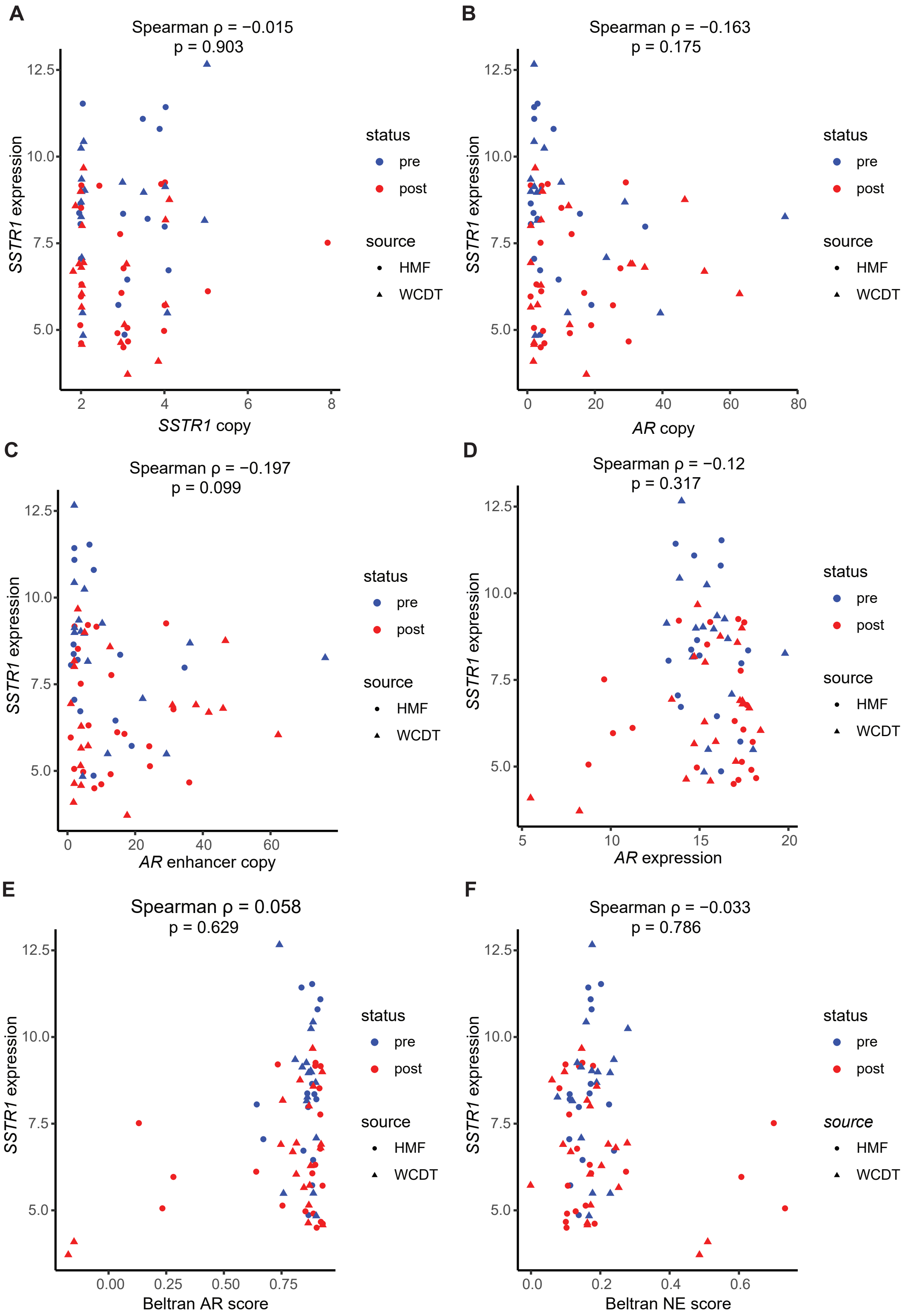

A
Spearman ρ = −0.015
p = 0.903
SSTR1 expression
SSTR1 copy
B
Spearman ρ = −0.163
p = 0.175
SSTR1 expression
AR copy
C
Spearman ρ = −0.197
p = 0.099
SSTR1 expression
AR enhancer copy
D
Spearman ρ = −0.12
p = 0.317
SSTR1 expression
AR expression
E
Spearman ρ = 0.058
p = 0.629
SSTR1 expression
Beltran AR score
F
Spearman ρ = −0.033
p = 0.786
SSTR1 expression
Beltran NE score
status
pre
post
source
HMF
WCDT

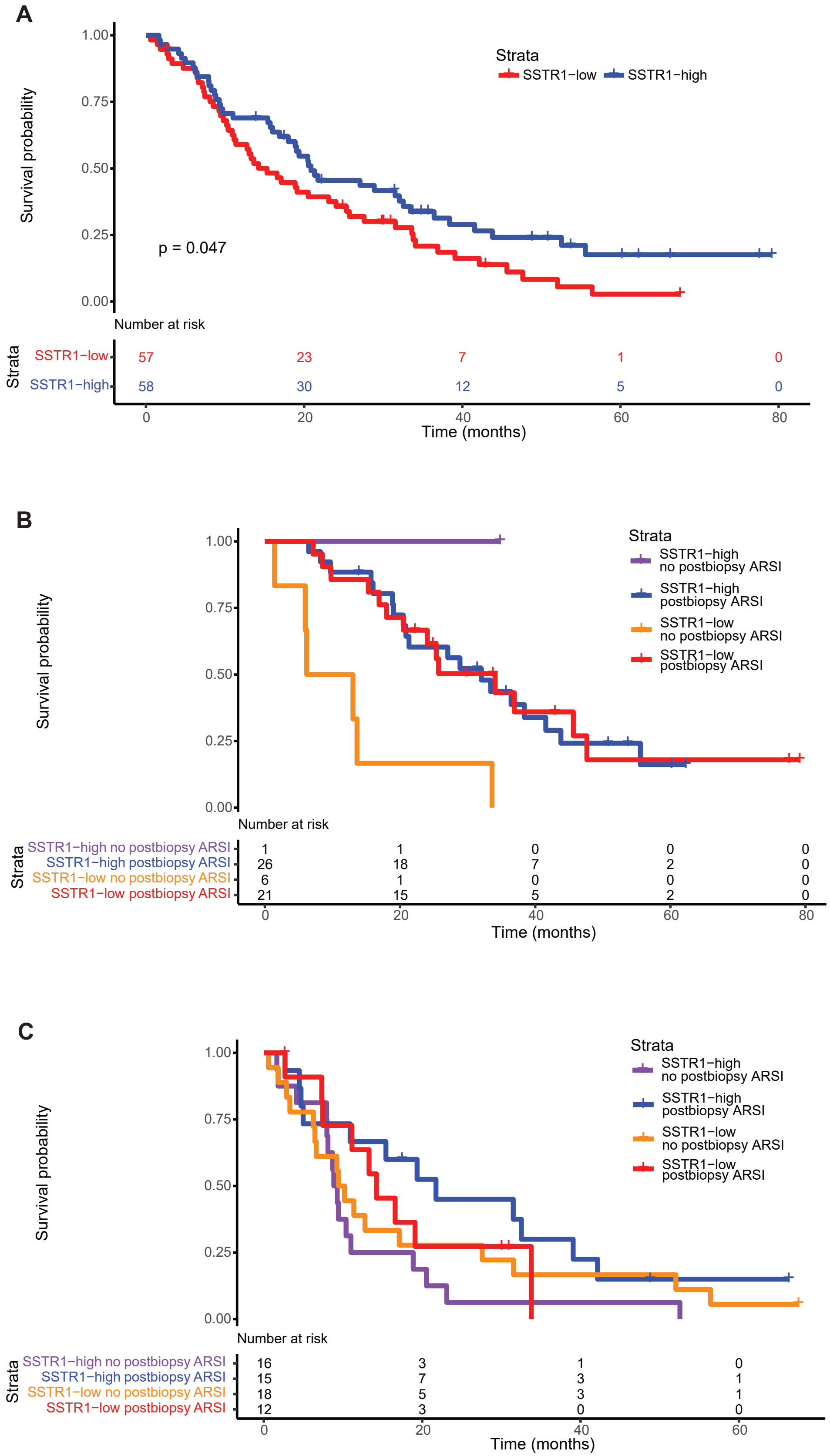
A
Survival probability
1.00
0.75
0.50
0.25
0.00
Strata
SSTR1−low
SSTR1−high
p = 0.047
Number at risk
Strata
SSTR1−low 57 23 7 1 0
SSTR1−high 58 30 12 5 0
0
20
40
60
80
Time (months)
B
Survival probability
1.00
0.75
0.50
0.25
0.00
Strata
SSTR1−high no postbiopsy ARSI
SSTR1−high postbiopsy ARSI
SSTR1−low no postbiopsy ARSI
SSTR1−low postbiopsy ARSI
Number at risk
Strata
SSTR1−high no postbiopsy ARSI 1 1 0 0 0
SSTR1−high postbiopsy ARSI 26 18 7 2 0
SSTR1−low no postbiopsy ARSI 6 1 0 0 0
SSTR1−low postbiopsy ARSI 21 15 5 2 0
0
20
40
60
80
Time (months)
C
Survival probability
1.00
0.75
0.50
0.25
0.00
Strata
SSTR1−high no postbiopsy ARSI
SSTR1−high postbiopsy ARSI
SSTR1−low no postbiopsy ARSI
SSTR1−low postbiopsy ARSI
Number at risk
Strata
SSTR1−high no postbiopsy ARSI 16 3 1 0
SSTR1−high postbiopsy ARSI 15 7 3 1
SSTR1−low no postbiopsy ARSI 18 5 3 1
SSTR1−low postbiopsy ARSI 12 3 0 0
0
20
40
60
Time (months)

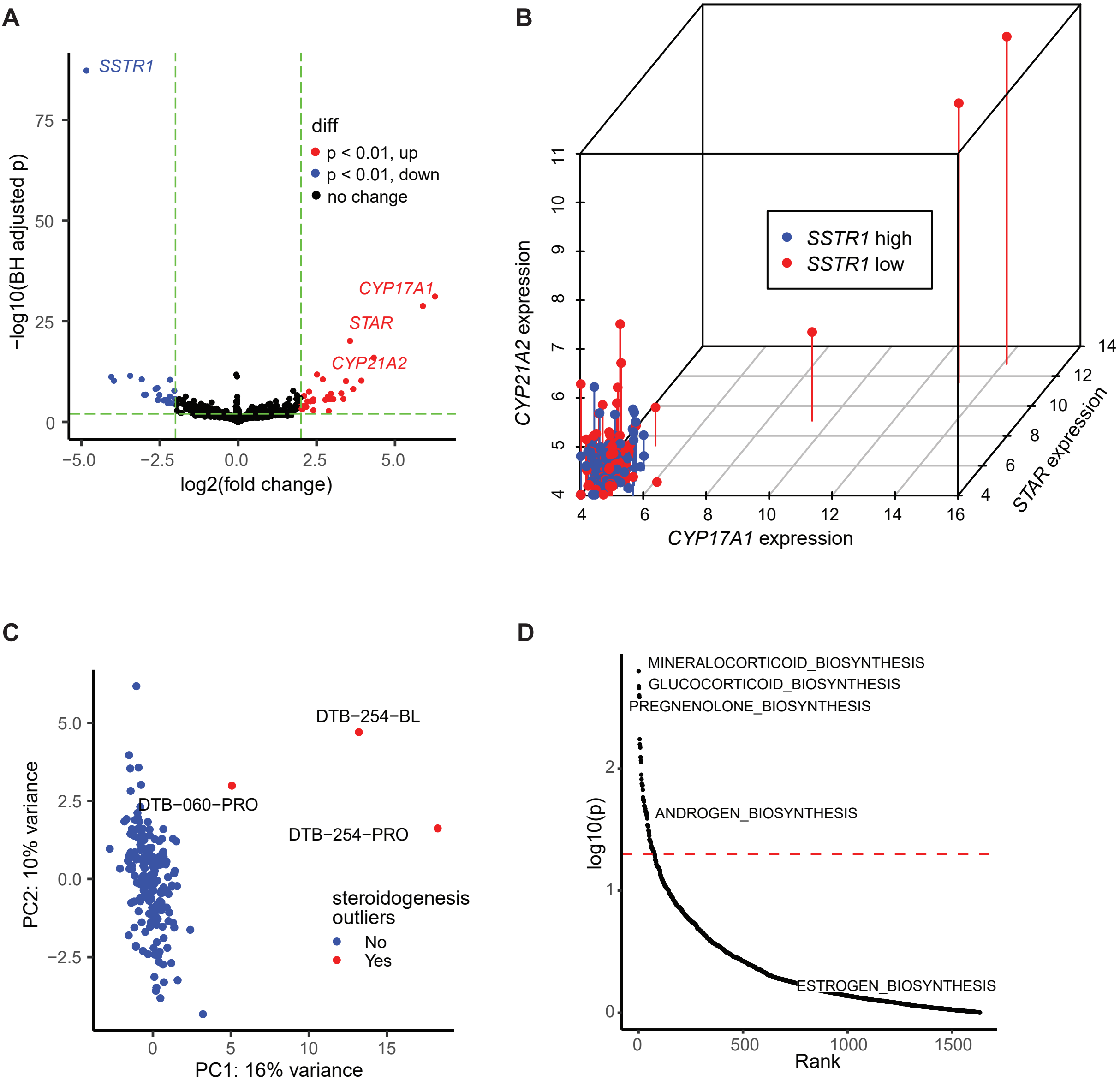
A
SSTR1
75
50
25
0
−log10(BH adjusted p)
diff
p < 0.01, up
p < 0.01, down
no change
CYP17A1
STAR
CYP21A2
−5.0
−2.5
0.0
2.5
5.0
log2(fold change)
B
11
10
9
8
7
6
5
4
CYP21A2 expression
SSTR1 high
SSTR1 low
4
6
8
10
12
14
16
CYP17A1 expression
14
12
10
8
6
4
STAR expression
C
5.0
2.5
0.0
−2.5
PC2: 10% variance
DTB−254−BL
DTB−060−PRO
DTB−254−PRO
steroidogenesis
outliers
No
Yes
0
5
10
15
PC1: 16% variance
D
MINERALOCORTICOID_BIOSYNTHESIS
GLUCOCORTICOID_BIOSYNTHESIS
PREGNENOLONE_BIOSYNTHESIS
ANDROGEN_BIOSYNTHESIS
ESTROGEN_BIOSYNTHESIS
2
1
0
log10(p)
0
500
1000
1500
Rank

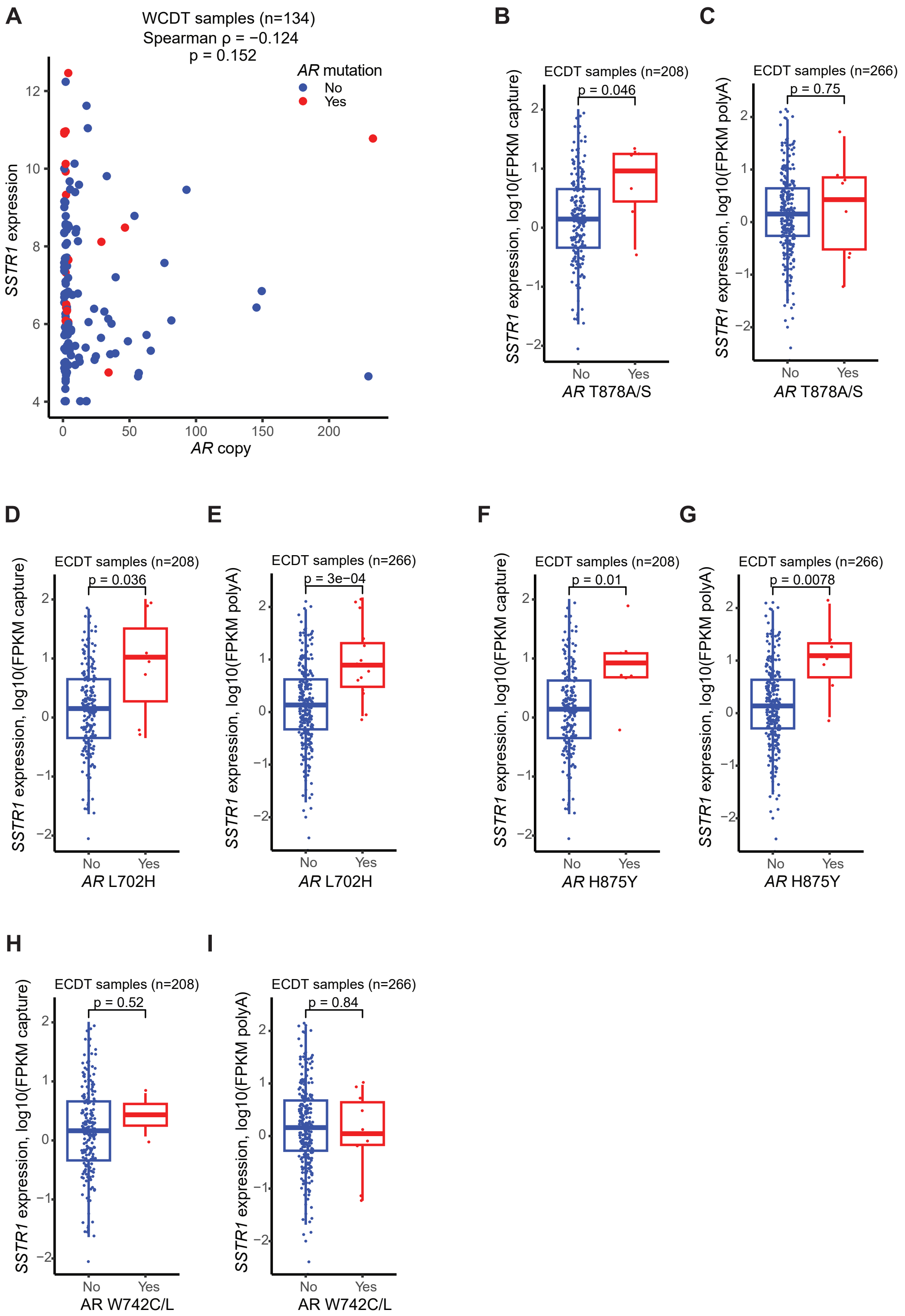

A
WCDT samples (n=134)
Spearman ρ = −0.124
p = 0.152
AR mutation
No
Yes
SSTR1 expression
AR copy
B
ECDT samples (n=208)
p = 0.046
SSTR1 expression, log10(FPKM capture)
AR T878A/S
C
ECDT samples (n=266)
p = 0.75
SSTR1 expression, log10(FPKM polyA)
AR T878A/S
D
ECDT samples (n=208)
p = 0.036
AR L702H
E
ECDT samples (n=266)
p = 3e-04
AR L702H
F
ECDT samples (n=208)
p = 0.01
AR H875Y
G
ECDT samples (n=266)
p = 0.0078
AR H875Y
H
ECDT samples (n=208)
p = 0.52
AR W742C/L
I
ECDT samples (n=266)
p = 0.84
AR W742C/L

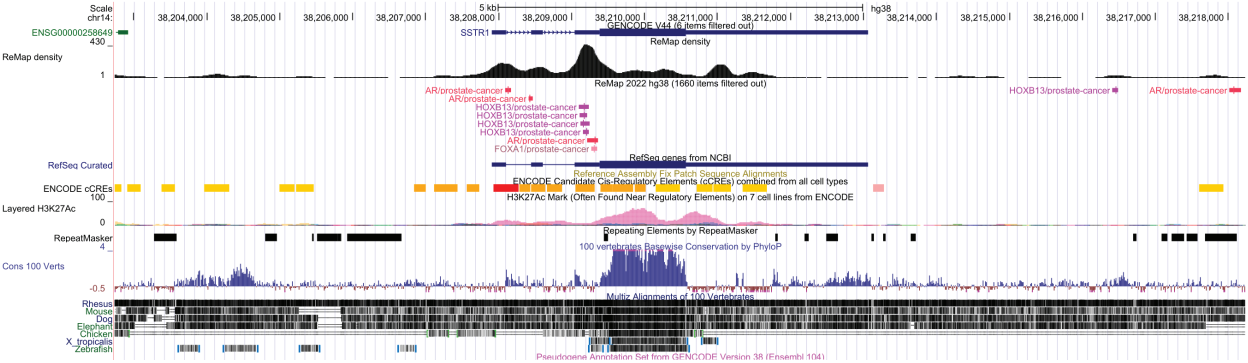

Scale
chr14:
5 kb
hg38
38,204,000
38,205,000
38,206,000
38,207,000
38,208,000
38,209,000
38,210,000
38,211,000
38,212,000
38,213,000
38,214,000
38,215,000
38,216,000
38,217,000
38,218,000
GENCODE V44 (6 items filtered out)
ENSG00000258649
SSTR1
ReMap density
430
ReMap density
1
ReMap 2022 hg38 (1660 items filtered out)
AR/prostate-cancer
AR/prostate-cancer
HOXB13/prostate-cancer
HOXB13/prostate-cancer
HOXB13/prostate-cancer
HOXB13/prostate-cancer
AR/prostate-cancer
FOXA1/prostate-cancer
HOXB13/prostate-cancer
AR/prostate-cancer
RefSeq genes from NCBI
RefSeq Curated
Reference Assembly Fix Patch Sequence Alignments
ENCODE Candidate Cis-Regulatory Elements (cCREs) combined from all cell types
ENCODE cCREs
100
H3K27Ac Mark (Often Found Near Regulatory Elements) on 7 cell lines from ENCODE
Layered H3K27Ac
0
Repeating Elements by RepeatMasker
RepeatMasker
4
100 vertebrates Basewise Conservation by PhyloP
Cons 100 Verts
-0.5
Multiz Alignments of 100 Vertebrates
Rhesus
Mouse
Dog
Elephant
Chicken
X_tropicalis
Zebrafish
Pseudogene Annotation Set from GENCODE Version 38 (Ensembl 104)

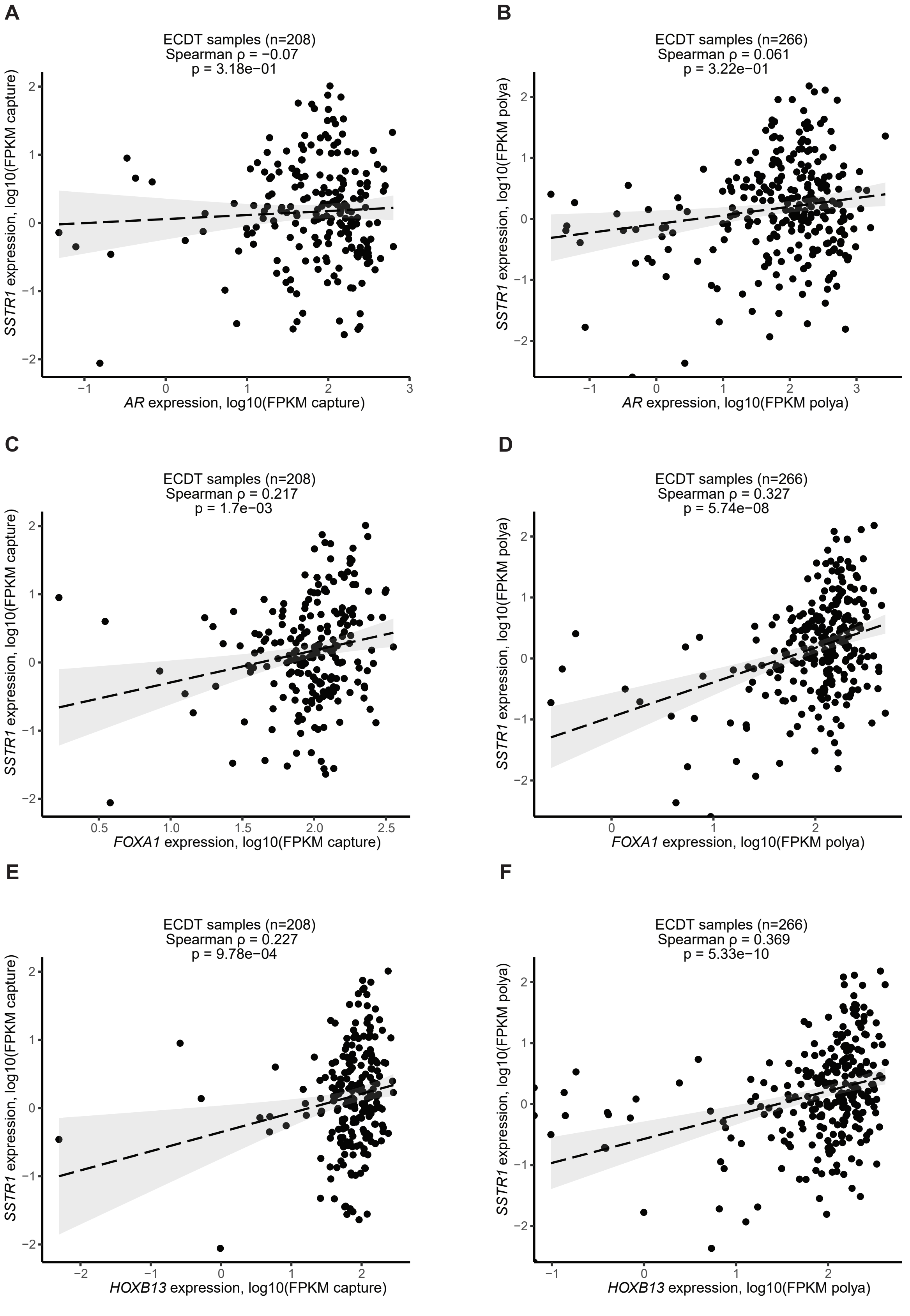
A
ECDT samples (n=208)
Spearman ρ = −0.07
p = 3.18e−01
SSTR1 expression, log10(FPKM capture)
AR expression, log10(FPKM capture)
B
ECDT samples (n=266)
Spearman ρ = 0.061
p = 3.22e−01
SSTR1 expression, log10(FPKM polya)
AR expression, log10(FPKM polya)
C
ECDT samples (n=208)
Spearman ρ = 0.217
p = 1.7e−03
SSTR1 expression, log10(FPKM capture)
FOXA1 expression, log10(FPKM capture)
D
ECDT samples (n=266)
Spearman ρ = 0.327
p = 5.74e−08
SSTR1 expression, log10(FPKM polya)
FOXA1 expression, log10(FPKM polya)
E
ECDT samples (n=208)
Spearman ρ = 0.227
p = 9.78e−04
SSTR1 expression, log10(FPKM capture)
HOXB13 expression, log10(FPKM capture)
F
ECDT samples (n=266)
Spearman ρ = 0.369
p = 5.33e−10
SSTR1 expression, log10(FPKM polya)
HOXB13 expression, log10(FPKM polya)

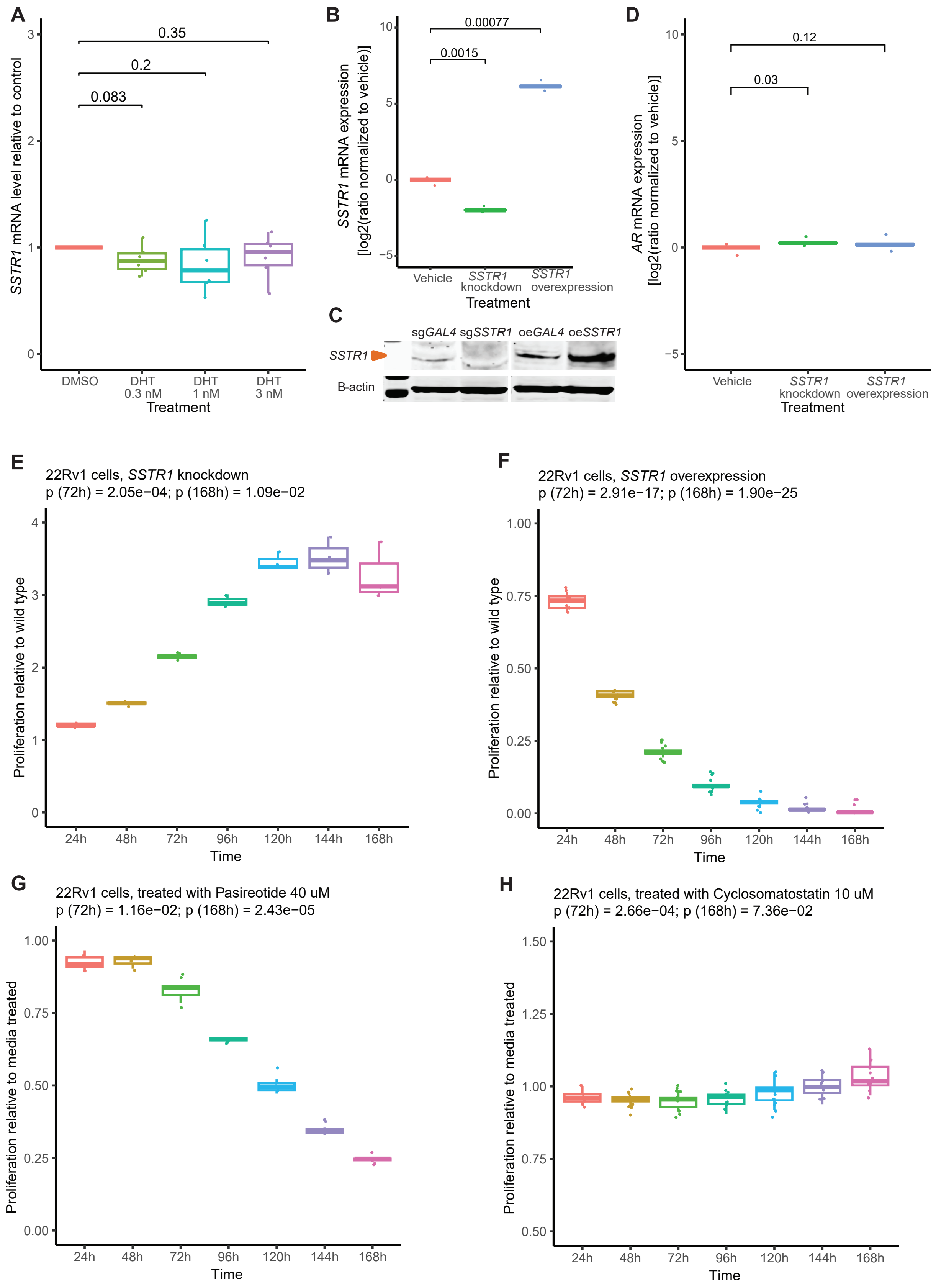
A
SSTR1 mRNA level relative to control
0.35
0.2
0.083
DMSO
DHT 0.3 nM
DHT 1 nM
DHT 3 nM
Treatment
B
SSTR1 mRNA expression [log2(ratio normalized to vehicle)]
0.00077
0.0015
Vehicle
SSTR1 knockdown
SSTR1 overexpression
Treatment
C
sgGAL4 sgSSTR1 oeGAL4 oeSSTR1
SSTR1
B-actin
D
AR mRNA expression [log2(ratio normalized to vehicle)]
0.12
0.03
Vehicle
SSTR1 knockdown
SSTR1 overexpression
Treatment
E
22Rv1 cells, SSTR1 knockdown
p (72h) = 2.05e−04; p (168h) = 1.09e−02
Proliferation relative to wild type
24h 48h 72h 96h 120h 144h 168h
Time
F
22Rv1 cells, SSTR1 overexpression
p (72h) = 2.91e−17; p (168h) = 1.90e−25
Proliferation relative to wild type
24h 48h 72h 96h 120h 144h 168h
Time
G
22Rv1 cells, treated with Pasireotide 40 uM
p (72h) = 1.16e−02; p (168h) = 2.43e−05
Proliferation relative to media treated
24h 48h 72h 96h 120h 144h 168h
Time
H
22Rv1 cells, treated with Cyclosomatostatin 10 uM
p (72h) = 2.66e−04; p (168h) = 7.36e−02
Proliferation relative to media treated
24h 48h 72h 96h 120h 144h 168h
Time

Supplemental Figure 1
Genomic landscape of the 45 paired mCRPC biopsies obtained before and after ARSI therapy. Samples are split by pair (patient), and patients are sorted by the upstream *AR* enhancer (reported by Quigley et al., 2018) copy number of the resistant tumor post-ARSI.

Supplemental Figure 2
Patient-level summary of the 45 paired mCRPC biopsies stratified by sample source (WCDT vs HMF) and ARSI treatment status (pre vs post). (**A**) Tumor purity. (**B**) Tumor ploidy. (**C**) Tumor mutation burden (TMB) per Mb. (**D**) Structural variant (SV) burden. **HMF**: Hartwig Medical Foundation. **WCDT**: West Coast Dream Team. P-values were calculated using two-sided paired Wilcoxon tests.

Supplemental Figure 3
For variants detected both pre-ARSI and post-ARSI in each patient, change in variant allele frequency (VAF) is highly correlated with change in estimated tumor purity. Data represent all variants detected as such in the 45 paired mCRPC biopsies.

Supplemental Figure 4
Both *AR* and the upstream *AR* enhancer acquire further copy gains post-ARSI. Figure panels show the copy number of *AR* (**A**) and the upstream *AR* enhancer (reported by Quigley et al., 2018, **B**), respectively, of the 45 paired mCRPC biopsies stratified by sample source (HMF vs WCDT).

Supplemental Figure 5
***AR* mutations and *AR* gene/enhancer amplifications are not significantly associated with the duration of response to ARSI therapy (i.e., abiraterone or enzalutamide in the current study).** (**A**-**C**) *AR*-related genetic alterations detected post-ARSI. (**D**-**F**) *AR*-related genetic alterations detected pre-ARSI.

Supplemental Figure 6
***AR* gene or enhancer-amplified mCRPC tumors without tandem duplications detected as the underlying events tend to have higher copy numbers, suggesting that extrachromosomal DNA may be a potential mechanism driving the amplifications.** (**A**) *AR* copy number is significantly higher in such tumors. (**B**) *AR* upstream enhancer copy number shows a similar trend.

Supplemental Figure 7
The fractions of AR-V7 or full-length AR (AR-FL) are not significantly different in mCRPC tumors before and after ARSI therapy, but some tumors show large numerical changes. (**A**-**B**) AR-V7 and AR-FL fractions in 31 pre-ARSI and 40 post-ARSI samples. (**C**-**D**) AR-V7 and AR-FL fractions in 31 pairs.

Supplemental Figure 8
The androgen biosynthesis pathway activity assessed by RNA-seq is not significantly different in mCRPC tumors before and after ARSI therapy but shows a decreasing trend in the three tumors switching to an AR-independent phenotype as reflected by a high NE score (>0.4 as reported by Beltran et al., 2016). (**A**-**B**) Androgen biosynthesis activity in all samples and the 31 pairs, respectively. (**C**-**D**) Androgen biosynthesis activity in the 28 pairs with post-ARSI Beltran NE score ≤0.4 and the 3 pairs >0.4, respectively.

Supplemental Figure 9
Copy number of C4 is significantly increased post-ARSI.

Supplemental Figure 10
AR is not predicted to bind to C4. A survey of cistromeDB nominates AR as the top transcription factor binding to C1-C3 based on the extent of overlaps with publicly available ChIP-seq datasets (**A**-**C**). In contrast, there is a lack of AR binding predicted in C4 (**D**).

Supplemental Figure 11
FOXA1 but not steroid receptors are predicted to bind to C4. Sets of assay for transposase-accessible chromatin with sequencing (ATAC-seq) peaks of human mCRPC (data from Tang et al., 2022) overlapping C1-C3 (**A**) and C4 (**B**) harbor distinct transcription factor (TF) binding motifs based on the Analysis of Motif Enrichment. While steroid receptors are predicted to bind to C1-C3, a distinct set of TFs are predicted to bind to C4, including FOXA1.

Supplemental Figure 12
***FOXA1* and *AR* are co-expressed in WCDT samples.**

Supplemental Figure 13
FOXA1 ChIP-seq peaks are observed in C4 in multiple prostate cancer cell lines, primary prostate cancer, and mCRPC (data from ReMap2022).

Supplemental Figure 14
Removing the batch effect due to sample source (HMF versus WCDT) in RNA-seq analysis. (**A**) Principal component analysis (PCA) before correcting the RNA-seq data for sample source. (**B**) PCA after correcting for sample source.

Supplemental Figure 15
Variability of gene expression increases post-ARSI across all 22 genes used for the molecular subtyping of mCRPC by Labrecque et al., 2019. Var(post) and Var(pre) denote the variance of gene expression in post-ARSI and pre-ARSI samples, respectively.

Supplemental Figure 16
Measuring mCRPC transcriptional phenotypes using the subtyping scheme reported by Tang et al., 2022 (defined by transcription factor drivers) further highlights tumor heterogeneity associated with ARSI therapy. (**A**-**D**) The four subtype scores (AR, WNT, NEPC, and SCL) in 31 pairs before and after ARSI therapy. No significant difference was observed, but some pairs showed dramatic changes after ARSI therapy. (**E**) The four subtype scores seem to measure different aspects of tumor transcriptional profiles, as reflected by a frequent lack of correlation between changes in these scores. (**F**) Principal component analysis using the changes in the four subtype scores highlights four post-ARSI outliers. (**G**-**L**) Pairwise scatter plots of the four subtype scores in 31 pairs with RNA-seq data. Pairs are labeled with directed linear segments (pre-ARSI → post-ARSI).

Supplemental Figure 17
ChIP-seq signals of FOXA1 observed in the promoter of *LMO3* in mCRPC are distinct from those in primary prostate cancer (ReMap2022; Hammal et al., 2022). *LMO3* encodes a neuroendocrine transcription factor and is the most significantly upregulated gene in the unpaired RNA-seq analysis of mCRPC biopsies.

Supplemental Figure 18
FGFR pathways are among the most upregulated post-ARSI in RNA-Seq analysis. (**A**) Volcano plot of unpaired RNA-seq analysis of Reactome pathways highlights increased activities of multiple FGFR pathways post-ARSI. (**B**) Component genes of the Reactome FGFR3 and FGFR2 pathways are shown in the Venn diagram.

Supplemental Figure 19
***SSTR1* is highly expressed in primary prostate cancer compared to other cancer types** (TCGA data, cancer types sorted by median *SSTR1* mRNA).

Supplemental Figure 20
***SSTR1* is expressed in mCRPC cells rather than immune cells in the tumor** (data from He et al., 2021 and visualized using the Single Cell Portal).

Supplemental Figure 21
***SSTR1* expression in mCRPC biopsies.** *SSTR1* expression is not associated with *SSTR1* copy number (**A**), *AR* copy number (**B**), the upstream *AR* enhancer copy number (**C**), *AR* mRNA level (**D**), Beltran AR score (**E**), or Beltran neuroendocrine (NE) score (**F**).

Supplemental Figure 22
Tumor *SSTR1* mRNA as related to survival in a retrospective analysis of mCRPC patients. (**A**) Higher *SSTR1* expression is associated with better overall survival in WCDT samples. (**B** and **C**) Survival curves stratified by *SSTR1* expression status (high versus low) and post-biopsy ARSI exposure (yes versus no) in patients with ARSI-naïve and ARSI-exposed mCRPC tumors, respectively, in WCDT.

Supplemental Figure 23
Excessive steroidogenesis is associated with ARSI resistance in a subset of *SSTR1*-low mCRPC. (**A**) Differential gene expression analysis contrasting *SSTR1*-high versus *SSTR1*-low tumors highlights upregulation of steroidogenesis genes including *CYP17A1*, *STAR*, and *CYP21A2*, in *SSTR1*-low mCRPC, driven by three outlier samples (**B**). (**C**) Principal component analysis using expression data of 24 genes in the five steroidogenesis pathways (mineralocorticoid, glucocorticoid, androgen, estrogen, and pregnenolone) confirms that the three *SSTR1*-low tumors are indeed outliers in steroidogenesis activity. (**D**) These three tumors maintain a more pronounced activity of mineralocorticoid, glucocorticoid, and pregnenolone biosynthesis rather than androgen biosynthesis compared to the other tumors.

Supplemental Figure 24
***SSTR1* mRNA as related to *AR* mutations.** (**A**) *SSTR1* expression is not associated with *AR* copy number in 134 WCDT samples with both WGS and RNA-seq data available. (**B-I**) In ECDT, the presence of an *AR* hotspot mutation such as T878A/S, L702H, and H875Y, but not W742C/L, is associated with increased *SSTR1* expression. FPKM: Fragments Per Kilobase of transcript per Million mapped reads.

Supplemental Figure 25
ChIP-seq signals of AR, FOXA1, and HOXB13 are observed in mCRPC patient-derived xenografts (PDXs) overlapping with exon 3 of *SSTR1* (ReMap2022; Hammal et al., 2022).

Supplemental Figure 26

***FOXA1* and *HOXB13*, but not *AR*, are co-expressed with *SSTR1* in ECDT samples.** Analyses are stratified by RNA-seq method (“capture” vs “polyA”). FPKM: Fragments Per Kilobase of transcript per Million mapped reads.

Supplemental Figure 27

SSTR1 is anti-proliferative in mCRPC and amenable to pharmacological intervention. (**A**) *SSTR1* mRNA level, as assessed by reverse transcription-quantitative polymerase chain reaction (RT-qPCR), did not change after 48 hours of dihydrotestosterone (DHT) treatment in 22Rv1 cells. (**B** and **C**) RT-qPCR and western blot validation, restively, of *SSTR1* stable knockdown assay using Clustered Regularly Interspaced Palindromic Repeats interference (CRISPRi) and *SSTR1* stable overexpression assay in 22Rv1 cells. All bands indicate ~80kDa as validated by the vendor (Cell Signaling Technology). The lanes were run on the same gel but were noncontiguous. (**D**) *AR* mRNA expression, as assessed by RT-qPCR, did not seem to change significantly with *SSTR1* knockdown or overexpression in 22Rv1 cells. (**E**) *SSTR1* knockdown significantly increased the proliferation of 22Rv1 cells using the IncuCyte cell count proliferation assay, and *SSTR1* overexpression had the opposite effect (**F**). (**G**) Pasireotide, the only FDA-approved drug with SSTR1 agonist activity, significantly inhibited the proliferation of 22Rv1 cells, whereas the SSTR1 antagonist cyclosomatostatin did not seem to have a substantive effect (**H**). P-values were calculated using Student's t-test.
