# Supplementary material for: Genomic and transcriptomic features of androgen receptor signaling inhibitor resistance in metastatic castration-resistant prostate cancer
Source: J Clin Invest. 2024 Aug 13;134(19):e178604. doi: 10.1172/JCI178604 (PMC11444163; doi:10.1172/JCI178604)
Supplement: Unedited blot and gel images [file jci-134-178604-s010.pdf]

Full unedited gel for Supplemental Figure 27C

Acquisition 07

Image Report Date Jul 11, 2024, 10:04:44 AM PDT

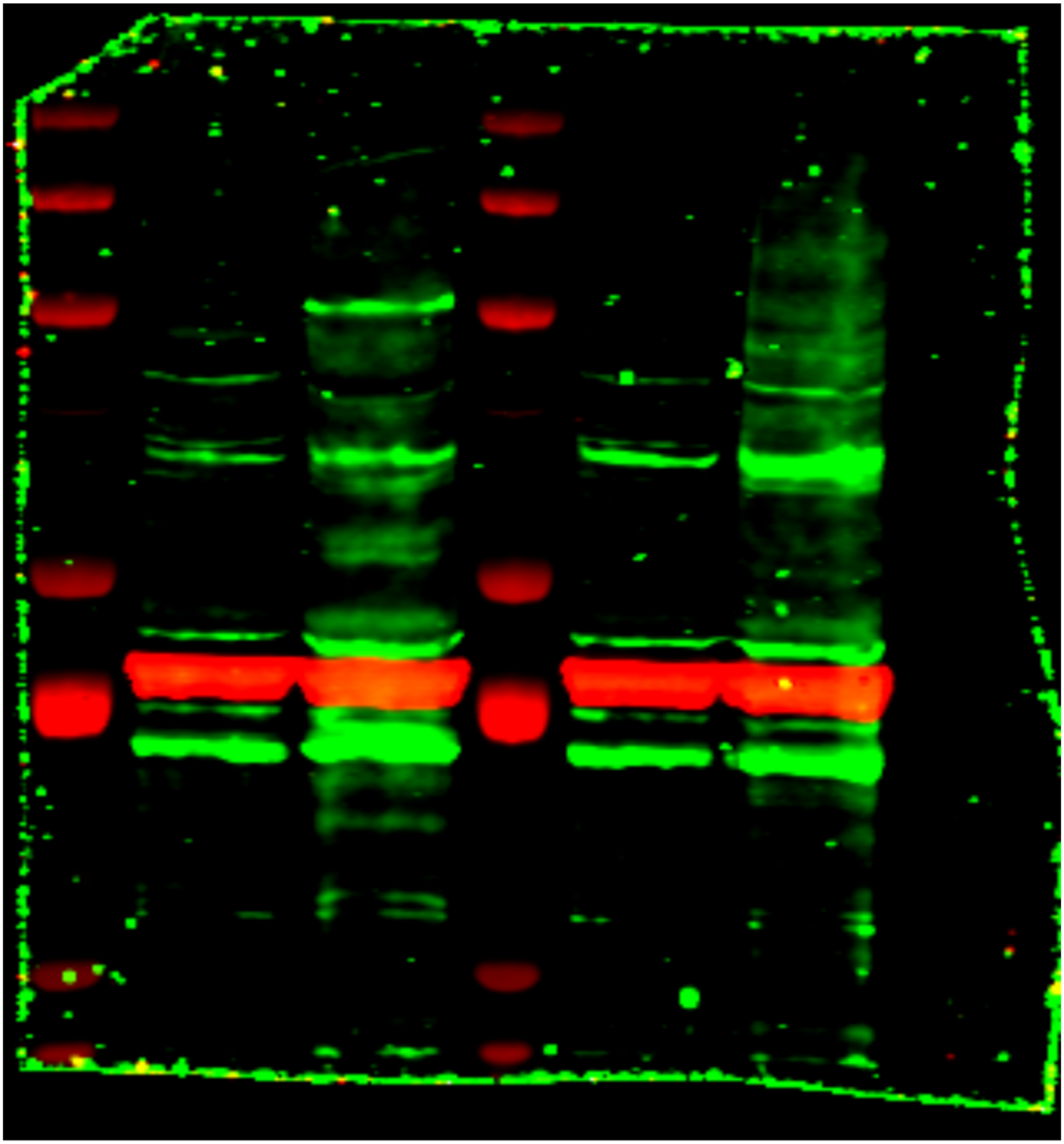

SSTR1 band 1

SSTR1 band 2

b-actin

700	Min: 18.3	Max: 161
800	Min: 6.85	Max: 14.6

General Information

Acquire Date/Time
Jul 10, 2024, 12:04:30 PM PDT

Assay
Membrane

Comments
Not specified

Image ID
0000612

Specimen ID
Not specified

User
Tatyanah

Imager Information

Imager Model
Odyssey CLx

Imager Name
LICOR-CLX-1750

Intensities
auto

Resolution
169 µm

Focus Offset
0.00 mm

Scan Area (cm)
X: 0.1 Y: 0.1 W: 5.4 H: 5.8

Position
1

Source Information

Acquisition Software
LI-COR Acquisition v2.0.0.86

Acquire Data Folder
D:\Licor
